# Older men and loneliness: a cross-sectional study of sex differences in the English Longitudinal Study of Ageing

**DOI:** 10.1186/s12889-024-17892-5

**Published:** 2024-02-02

**Authors:** John Ratcliffe, Paul Galdas, Mona Kanaan

**Affiliations:** 1 Department of Health Sciences, Seebohm Rowntree Building, University of York, Heslington, York YO10 5DD UK; 2 Sheffield Hallam University, College of Health, Wellbeing, and Life Sciences, Robert Winston Building, Sheffield, S10 2BP UK

**Keywords:** Loneliness, Social isolation, Gerontology, Alcohol, Marriage, Masculinity

## Abstract

**Background:**

Research into men and masculinities suggests men may be more reluctant than women to state they are lonely, more reliant on partners/spouses and/or alcohol to tackle it, and that this may be a result of poorer social relationships. Ageing is often associated with loneliness, and research has indicated gendered results in older people, but existing evidence lacks generalisability and cultural context. This study tests hypotheses on sex differences in loneliness in older England-based men and women.

**Methods:**

We conducted a cross-sectional study using a sample of 6936 respondents aged 50 + from the English Longitudinal Study of Ageing (wave 8). Multiple imputation with chained equations was conducted to handle missing data. Multivariate regression was used to investigate the impact of sex on a direct question on loneliness whilst controlling for the University of California loneliness (UCLA) scale. Multivariate regression with interaction terms were used to examine sex differences in loneliness and alcohol consumption, partner status, and social relationships.

**Results:**

Older men were less likely than older women to state they are lonely even when controlling for UCLA score. Older men showed a greater association between loneliness and alcohol consumption, but only when measuring the number of units consumed in the last week, and not using a less precise measure of the past year. Older men who cohabited with a partner were less lonely than cohabiting older women, whereas previously married but not cohabiting older men were lonelier than their female counterparts. However, never married older men were less lonely than never married older women. Evidence was found to suggest older men’s worse friendships mediated this association, but social isolation and number of close relationships did not. Severe isolation predicted greater loneliness in older women, but not older men.

**Conclusions:**

Cultural ideals of masculinity and older men’s poorer quality friendships may explain their reluctance to directly state loneliness, greater dependency on partners/spouses, and use of alcohol. Severely isolated older men may under-report loneliness on the UCLA scale as well as a direct question.

**Supplementary Information:**

The online version contains supplementary material available at 10.1186/s12889-024-17892-5.

## Introduction

Conceptualisations of loneliness vary, yet most agree it is a subjective emotion related to poor social relationships [1–4]. It has been argued to be a growing problem both in the United Kingdom (UK) and worldwide [5], and the onset of Covid-19 pandemic increased these concerns [6]. Mental health research has highlighted the impact of masculinities on mental health [7], and loneliness has been cited as a particular problem in older people [8]. Qualitative work with older men has emphasised the embodied reformation of masculine ideals as vital to gendered experiences of ageing [9, 10], and masculinities have been placed as central to older men’s experiences of loneliness [11]. However, some widely held theoretical notions on gender and loneliness have not been empirically evidenced using England-based generalisable data.

Though some UK research has found older women to be lonelier than older men [8, 12, 13], these studies draw their data from a ‘direct’ question employing the word ‘lonely’. Rokach [14] and De Jong-Gierveld et al. [15] argue men may ‘understate’ loneliness on a direct question as it signifies a vulnerable and less masculine state. In Norway, Nicolaisen and Thorsen [16] compared a direct question to the De Jong-Gierveld loneliness scale and found older women are lonelier than older men on the former, but no statistically significant difference on the latter. Similarly, Steed et al. [17] found no evidence of a statistically significant sex difference in older Australian adults when examining the University of California, Los Angeles loneliness (UCLA) scale, yet women were significantly lonelier using a direct question. Despite the commonality of this theoretical notion, though, few studies interrogate it, and none, to our knowledge, have done so in England. Indeed, unlike Steed et al. [17], some UK work [18] has found women to show significantly higher odds of loneliness using the UCLA scale.

Studies have suggested people perceive alcohol abuse to be a male or masculine response to loneliness [19–21]. UK-based qualitative research has offered support for this. Ratcliffe et al. [22] found several men characterised their alcoholism as a masculine method of handling loneliness, and Hubach et al. [23] concluded young men self-medicated loneliness with drugs (including alcohol). In the UK, men drink significantly more alcohol than women, albeit this difference becomes smaller with age [24]. To our knowledge, though, no study has quantified whether loneliness shows a greater association with alcohol consumption in older men than it does in older women. It is therefore unclear whether older men are more likely to abuse alcohol when they are lonely than women are.

A wealth of research has indicated that older men’s loneliness depends more on whether they have a partner/spouse than women’s loneliness does [25–27]. Nowland et al. [28], in a study conducted in North-West England, found that loneliness in women was not dependent on relationship status, but men were lonelier and more sensitive to rejection if they were not in a relationship. However, little work has examined whether this sex difference is similarly true for people who have never married in comparison to those who have experienced marriage. Never married older people are theoretically more likely to prefer being single (given that they have never chosen to marry), and/or may have developed greater resilience, therefore this distinction requires investigating, particularly in older populations.

Qualitative work, including studies with older men in the UK, has suggested men are more reliant on partners/spouses for intimate companionship [29–32]. In these works, masculine cultures are said to eschew vulnerability, making it more difficult to form intimate relationships, rendering men reliant on a partner/spouse for social support and opportunities. Moreover, this worsens with age as masculine modes of forming social connections through work and physical activity are depleted [11, 30, 33–35]. However, while this is a theoretically plausible explanation of quantitative data indicating a greater association between partner status and loneliness in older men, to the best of our knowledge, it has not been interrogated on a larger scale.

Studies conducted in Northern Europe using the De Jong-Gierveld scale add further complexity. Many have found men display more ‘social’ loneliness, and women more ‘emotional’ loneliness [16, 26, 36]. Here, ‘social’ loneliness refers to the ‘absence of an engaging social network’, whereas ‘emotional’ loneliness is the ‘absence of a close emotional attachment’ ([37], p18−19). Men, then, may not lack intimate companionship, but social networks. Similarly, Takagi et al.’s [38] work on older Singaporeans found family networks had a greater impact on women’s loneliness, whereas some public networks had a greater impact on men. Cultural context may be vital—as Takagi et al. [38] conclude, sociocultural and demographic conditions among older Singaporean adults are unique to them. It is therefore vital to replicate and reassess data with regard to the context in which it is formed, particularly in studies of gender and loneliness.

Current literature has not offered generalisable evidence English older men are, as many posit, less lonely in response to a direct scale of loneliness than on the UCLA scale, and more likely to drink alcohol in response to loneliness. It is unclear whether work indicating older men are more reliant on partners/spouses similarly applies to these who have never married as it does to those who have experienced marriage, and whether work indicating non-spousal relationships has a greater impact on men is applicable to older England-based men and women. No research, to our knowledge, has offered generalisable evidence that poorer non-spousal relationships can explain men’s greater association between partner status and loneliness. The current study uses the English Longitudinal Study of Ageing (ELSA), a large England-based dataset of men and women aged 50 + , to conduct a cross-sectional study with one overarching objective, investigated via five hypotheses:

What sex differences in loneliness exist in England-based people aged 50+?

1. Men with equal scores of loneliness to women on an indirect scale of loneliness will be less likely to state they are lonely according to a direct question.
2. Loneliness will be more greatly associated with alcohol consumption in men than in women.
3. Men will show a greater increase in loneliness according to poorer social relationships than women.
4. Single men will show a greater increase in loneliness when compared to men with a partner than single women in comparison to women with a partner.
5. Men’s lower quality social relationships will mediate their greater reliance on partners for reducing the chances of loneliness.

## Methods

### Dataset and participants

The current study is a cross-sectional study using wave 8 of ELSA [39]. ELSA is a longitudinal dataset that began in 2002, after which data has been collected every two years. Eligible participants are aged 50 + , and reside in England, but not in an ‘institution’ (e.g., nursing home), at the time they are invited to take part [40]. Its sample is drawn from households who responded to the Health Survey for England either in 1998, 1999, or 2001, stratified by health authority and amount of non-manual socio-economic groups via a multi-stage probability sampling design [41]. To continue to provide representative cross-sectional data, new members were added to the study at waves 2, 4, 6, and 7 [42].

A cross-sectional study was conducted as the objective was to provide a current snapshot of sex differences in older people. Wave 8 was the most recent wave available when the study commenced. These data were collected between May 2016 and June 2017. This study used the core sample (7,223), minus 287 ‘proxy’ interviews, resulting in a sample size of 6,936. Proxy interviews used a designated individual to respond on behalf of a participant. They were removed as the personal nature of loneliness was considered ill-suited to being answered by a person who is not the actual respondent, particularly for the hypothesis investigating older men’s propensity to admit loneliness.

### Variables

The dependent variable for hypothesis 1 was direct measure of loneliness, and the dependent variables for hypothesis 2 measured alcohol consumption. Hypotheses 3 to 5 all used the dichotomised UCLA scale as the dependent variable. Key independent variables were sex, loneliness, partner status, and four measures of social relationships. This section details the variables and any transformations used.

### Loneliness

The study used three measures of loneliness: the three-item UCLA scale; a direct question; and another direct question focused on the last week. The UCLA scale was used in the majority of tests, the direct question was used to investigate whether older men show less loneliness than in response to this than in response to the UCLA scale, and the direct question in relation to the past week was used in models where key independent variables also referred to the past week. Despite the differences highlighted and investigated in the present study, research has found all three loneliness measures significantly correlate [12, 41, 43, 44].

The direct question asked ‘how often do you feel lonely?’ (hardly ever/never; some of the time; often). The direct question on loneliness in the past week asked ‘have you felt lonely much of the time in the last week (yes; no)’. It is included in ELSA as part of 8-item Centre for Epidemiologic Studies Depression (CES-D) Scale [45]. The three-item UCLA loneliness scale was formed by taking the revised 20-item version and identifying three factors: ‘I feel left out’; ‘I feel isolated’, and ‘I lack companionship’ [43]. This has shown good internal consistency and adequate concurrent and discriminant validity [46]. ELSA transformed these items into questions answerable as ‘hardly ever/never’, ‘some of the time’, or ‘often’:

1. How often do you feel you lack companionship?
2. How often do you feel isolated from others?
3. How often do you feel left out?

This produces a score between 3 and 9. When used as a dependent variable, this was dichotomised to overcome validity issues associated with the small range and skew of the score data [47, 48]. A score of 6 + is conventionally used to represent a score of ‘lonely’ [49, 50]. This has been criticised for arbitrarily creating a large distinction between scores of 5 and 6, in which 5 (not lonely) can be attained despite answering ‘often’ in one response, and 6 (lonely) can be attained by only answering ‘some of the time’ for all three items [41]. Nevertheless, dichotomisation allowed the study to produce interpretable models, therefore the current study will refer to a ‘score of lonely’ rather than an absolute ‘lonely’ to encapsulate this nuanced distinction.

### Alcohol consumption

Two variables were used to measure alcohol consumption in the current study. Both were self-report measures. The first asked:

How often have you had an alcoholic drink of any kind during the last 12 months (never—once every couple of months; once a month—twice a week; three—six days a week; almost every day or more)?

These four responses were a merged set of categories based on an eight-category response set used in ELSA, which were more difficult to present but provided similar results to the four-category variable.

The second variable was a measure of units of alcohol consumed in the past week. This was constructed from three variables: number of measures of spirit the respondent had consumed in the last week; number of glasses of wine (or similar drinks); and number of pints of beer or cider. Frischer et al. [51] state that one unit typically equals one measure of spirit (ABV 40%), 1/3 of a pint of beer (ABV 5%), or half a standard (175 ml) glass of red wine (ABV 12%). To calculate an estimate of the units of alcohol consumed, the number of pints were multiplied by 3, and the number of glasses of wine by 2, creating the final variable ‘estimated total number of units of alcohol consumed in the past week’.

### Partner status

Partner status consisted of three categories: cohabiting with a partner; never married and not cohabiting with a partner; previously married but not cohabiting with a partner. These were formed by combining and merging two variables: do you have a husband, wife or partner with whom you live (yes/no); and marital status (in a first marriage; in a second or later marriage; never married; separated; divorced; widowed). ‘Cohabiting with a partner’ represents all who answered ‘yes’ to the former (regardless of marital status). ‘Never married and not cohabiting with a partner’ represents those who answered ‘no’ and ‘never married’. ‘Previously married but not cohabiting with a partner’ represents ‘no’ plus any marital status except for ‘never married’.

### Social relationships

The measures of social relationships used in the current study are an ‘Indicator of Severe Isolation (ISI)’, an ‘Index of Close Relationships (ICR)’, an ‘Indicator of Any close Relationships (IAC)’, and a ‘Perception of Friendship Relationships (PFR)’ score. The ISI is based on Shankar et al.’s [52] index of social isolation. In the present study, it consisted of a dichotomous variable representing ‘severely isolated’ or ‘not severely isolated’. However, contact with partners/spouses was not included as this would prevent the study from investigating the impact of social isolation on men’s reliance on partners/spouses. Respondents received a score of ‘severely socially isolated’ if they indicated all the following (‘contact' refers to face-to-face, telephone, or written/email contact):

- Less than monthly contact with children.
- Less than monthly contact with friends.
- Less than monthly contact with other family (not spouses/partners or children).
- No participation in any organisations, religious groups, or committees.
- Not a member of a gym or sports club.

The ICR is based on a scale developed by Valtorta [41], with reference to partners/spouses removed. It is formed by adding together responses to the following three questions:

- Number of children with whom the respondent has a close relationship.
- Number of other family members with whom the respondent has a close relationship.
- Number of friends someone has a close relationship with.

The IAC is a dichotomised version of the ICR. People with a score of zero are coded as ‘no close relationships’, and people who have a score of one or more are coded as having ‘at least one close relationship’. This was formed as the difference between no close relationships, and one or more, was considered potentially more meaningful than a linear measurement.

PFR was derived from seven items asking about people’s perceived relationships with their friends:

- How much do they really understand the way you feel about things?
- How much can you rely on them if you have a serious problem?
- How much can you open up to them if you need to talk about your worries?
- How much do they criticise you?
- How much do they let you down when you are counting on them?
- How much do they get on your nerves?
- How often do they make too many demands on you?

These are answerable as ‘a lot’, ‘some’ ‘a little’ and ‘not at all’. For the positively worded items, ‘a lot’ was scored as 4, and ‘not at all’ as 1, and vice-versa for the negatively items. This creates a score between 7 and 28, where 7 represents an extremely poor perception of their friendships.

### Sex

Sex is answerable as ‘male’ or ‘female’. ELSA does not provide any further information.

### Covariates

A relatively large number of covariates were employed as each has been evidenced to impact loneliness or response rates in ELSA. Each covariate, and a reference to literature that found it to impact loneliness/response rates, is listed below.

- Ethnicity [22, 53, 54] (white; none-white).
- Age [8] at wave 8 (capped at 90 +)
- Employment status [55] (employed; self-employed; retired; permanently sick or disabled; looking after home or family; other)
- Whether has a long-standing and limiting illness [30, 35] (yes; no)
- Whether difficult walking 1/4 mile (0.4km) unaided [30, 35] (none; some; much; can’t)
- Wealth [56, 57]. Incorporates all owned assets, including properties, businesses, and savings, minus debt, at a ‘benefit unit’ level (the extent to which the respondent has access to the wealth). See Banks et al. [58] for details.
- Income [56, 57]. Measured as weekly, from any source, benefit unit level [58].
- Region [42] (Southern; Midlands; Northern/rest of UK)
- Education [59] (less than GCSE/equivalent/foreign qualification; GCSE/A-level/equivalent; higher than A-level). GCSEs are usually taken aged 16, and A-levels aged 18.

### Missing data analysis and imputation

SPSS version 25 [60] was used to investigate whether missing data would significantly influence the size and significance of the hypothesis tests. Univariate missingness rates were examined, and Little’s missing completely at random (MCAR) test was conducted [61]. Little’s MCAR test suggested the data were not MCAR (*P* < 0.001), and 30 of the 45 variables used in the final models had a missingness rate of 10% or more (additional file 1), suggesting a problematic missingness rate [62]. To account for this, Multiple imputation with Chained Equations (MICE) was conducted in Stata version 16 [63]. Predictive mean matching, with 10 nearest neighbours, was used for continuous variables [64], and augmentation was used on categorical variables [65]. A burnin of 20 was specified. Twenty-five datasets were imputed, and the pooled estimates used for analysis [66]. The do file for imputation is in additional file 2.

### Statistical analysis

Analyses were conducted in SPSS version 25 [60]. Findings using the pooled means and listwise deletion were computed for each test. The mean and standard error (SE) were computed for all continuous variables, and the numerical total (N) and within category percentages (%) were generated for categorical variables. Summaries for both the overall data, and the data disaggregated by sex, were computed. To add context to men’s and women’s odds of loneliness, an independent-samples T-test was conducted to compare men’s and women’s unadjusted UCLA scores.

Twelve regression models were constructed to test the five hypotheses. Details of each model, including the dependent and independent variables, and how they investigate the hypotheses, are in Table 1. The process of assumption testing and transformations that led to each model is in additional file 3. Hypothesis 2 encompassed two models as the measure of the past year doesn’t record how much alcohol was consumed on each occasion, whereas the measure of alcohol consumed in the last week does, but only refers to one week. Hypotheses 3 and 5 were both investigated four times, using the ISI, ICR, IAC, and PFR, as the ‘best’ measure could not be theoretically pinpointed. The ISI was the clearest measure of social isolation but didn’t consider the quality of the social relationships it records. The IAC and ICR incorporated a consideration of the quality of relationships, but include a significant focus on family relationships, therefore are less suited to investigating the hypothesis that men rely on partners/spouses due to poorer wider social relationships. The PFR was the best measure of the quality of non-family relationships, however it required excluding people with no friends. For hypotheses 2 to 5, a statistical significance of *P* < 0.05 for the coefficient of the interaction term was used to establish whether sex moderated the relationship. For hypothesis 5, the difference between model 4 and 5.1/5.2/5.3/5.4 was examined visually.

**Table 1:** Summary of statistical models and the hypothesis they investigate

*Model*	*Dependent variable*	*Regression type*	*Independent variables*	*Hypothesis*
1	How often lonely (rarely/never → sometimes → often)	Ordinal (Proportional odds)	3 item UCLA score, sex, covariates, partner status	With equal UCLA scores to women, men will be less likely to say they are lonely on a direct question
2.1	How often consumed alcohol in last 12 months	Multinomial	3-item UCLA score, sex, interaction terms for sex by 3-item UCLA score, covariates, partner status	Loneliness will result in a greater increase in unhealthy behaviour (alcohol consumption) in men than in women
2.2	Estimated units alcohol consumed in past week	Negative Binomial	Whether lonely in past 7 days, sex, interaction terms for sex by whether lonely in past 7 days, covariates, partner status	Loneliness will result in a greater increase in unhealthy behaviour (alcohol consumption) in men than in women
3.1	UCLA score (dichotomised)	Logistic	ISI, sex, interaction terms for sex by ISI, covariates, partner status	Men’s perception of their social network (ISI) is more important to whether they are lonely than it is for women
3.2	UCLA score (dichotomised)	Logistic	IAC, sex, interaction terms for sex by IAC, covariates, partner status	Men’s perception of their social network (IAC) is more important to whether they are lonely than it is for women
3.3	UCLA score (dichotomised)	Logistic	ICR, sex, interaction terms for sex by ICR, covariates, partner status	Men’s perception of their social network (ICR) is more important to whether they are lonely than it is for women
3.4	UCLA score (dichotomised)	Logistic	PFR, sex, interaction terms for sex by PFR, covariates, partner status	Men’s perception of their social network (PFR) is more important to whether they are lonely than it is for women
4	UCLA score (dichotomised)	Logistic	Partner status, sex, interaction terms for sex by partner status, covariates	Men will show greater dependence on having a cohabiting partner for not having a score of lonely than will women
5.1	UCLA score (dichotomised)	Logistic	ISI, partner status, sex, interaction terms for sex by partner status, covariates	Men will show a smaller association between partner status and loneliness than in model 4
5.2	UCLA score (dichotomised)	Logistic	IAC, partner status, sex, interaction terms for sex by partner status, covariates	Men will show a smaller association between partner status and loneliness than in model 4
5.3	UCLA score (dichotomised)	Logistic	ICR, partner status, sex, interaction terms for sex by partner status, covariates	Men will show a smaller association between partner status and loneliness than in model 4
5.4	UCLA score (dichotomised)	Logistic	PFR, partner status, sex, interaction terms for sex by partner status, covariates	Men will show a smaller association between partner status and loneliness than in model 4

## Results

All data presented below is taken from the imputed data, using pooled estimates. There were no large differences between the imputed and original data (Additional files 4, 5, 6, 7, 8, 9, 10, 11, 12, 13, 14 and 15).

### Descriptive statistics

Table 2 gives the descriptive statistics for the continuous and score variables, and Table 3 provides the statistics for the categorical and ordinal variables. The dataset is 44% male. Many more men were in a cohabiting relationship (76.0% compared to 60.3%), whilst many more women had experienced marriage but now lived without a partner (35.1% compared to 17.9%). 38.4% of men were educated beyond A-levels/equivalent, but just 26.1% of women were, and men had a greater mean income and wealth. Despite this, men drank an estimated mean of 11.5 units (SE 0.28) of alcohol in the last week, whereas women drank an estimated mean of just 5 (SE 0.14). Men were also almost twice as likely to drink ‘every day or more’ (men = 17.9%, women = 9.7%), whereas women were almost twice as likely to never or rarely drink (men = 22.7%, women = 41.0%).

**Table 2:** Means and standard errors for continuous variables by sex, using imputed data

Variable (min. – max. values if applicable)	Whole sample (*N* = 6936)		Women (*N* = 3900)		Men (*N* = 3036)	
	*Mean*	*SE*	*Mean*	*SE*	*Mean*	*SE*
3-item UCLA score (3–9)	4.15	0.02	4.23	0.03	4.05	0.03
Perception of friendships relationships (7–28) ^a^	23.68	0.04	24.27	0.05	22.90	0.06
Number of close relationships^b^	7.48	0.06	7.77	0.08	7.11	0.10
Units of alcohol in the last week	7.86	0.15	5.01	0.14	11.52	0.28
Age in years (50 – 90)	70.01	0.11	68.53	0.15	69.88	0.16
Wealth (benefit unit level)^c^	446633.23	9508.46	413428.51	6.90	489287.53	18101.75
Income (benefit unit level)^c^	555.88	5.47	514.82	9288.67	608.63	8.74

^a^ Imputation of whether a respondent has any friends was necessary, therefore N for perception of friendship relationships is based on a pooled mean. Whole sample *N* = 6453.6 Men’s *N* = 2770.2 Women’s *N* = 3683.4

^b^ Excluding partners/spouses

^c^ Benefit unit level’ refers to the extent to which the respondent has access to the wealth/income. Wealth refers to all owned assets, including properties, businesses, and savings, minus debt. Income refers to weekly income from any source. Details can be found in the ‘financial derived variables user guide’ [56]

**Table 3:** Cell counts for categorical variables, in total and by sex, using imputed data

Variable	Categories	Total		Women		Men	
		N	%	N	%	N	%
Total		6936	-	3900	-	3036	-
How often feels lonely	hardly ever/never	4808	69.3	2537	65.0	2271	74.8
	some of the time	1646	23.7	1043	26.7	604	19.9
	often	482	6.9	321	8.3	161	5.3
Have you felt lonely much of the time in the last week	yes	843	12.2	547	14.0	296	9.7
	no	6093	87.8	3353	86.0	2740	90.3
UCLA score (dichotomised)	not lonely (3–5)	5550	80.0	3049	78.2	2502	82.4
	lonely (6 +)	1385	20.0	851	21.8	534	17.6
Very socially isolated excluding partners (ISI)^a^	yes	61	0.9	19	0.5	42	1.4
	no	6820	99.1	3848	99.5	2972	98.6
Close relationships excluding partners (IAC)	has none	100	1.4	29.9	0.8	71	2.3
	has some	6835	98.6	3870	99.2	2965	97.6
How often had an alcoholic drink during last 12 months	never—once every couple of months	2288	32.9	1600	41.0	688	22.7
	once a month—twice a week	2426	34.9	1335	34.2	1091	35.9
	three—six days a week	1300	18.7	586	15.0	714	23.5
	almost every day or more	923	13.3	380	9.7	543	17.9
Partner status	cohabiting with a partner	4661	67.2	2353	60.3	2309	76.0
	experienced marriage not cohabiting	1893	27.3	1369	35.1	523	17.2
	never married not cohabiting	382	5.5	178	4.4	204	6.7
Ethnicity	white	6709	96.7	3777	96.8	2932	96.6
	non-white	227	3.3	123	3.2	104	3.4
Employment status	employed	1205	17.3	638	16.4	567	18.7
	self-employed	359	5.1	128	3.3	231	7.6
	retired	4796	69.1	2733	70.1	2063	68.0
	disabled	193	2.8	101	2.6	92	3.0
	looking after home or family	282	4.1	257	6.6	25	0.8
	other	101	1.5	43	1.1	58	1.9
Whether has a long-standing and limiting illness	yes	2511	36.2	1463	37.5	1047	34.5
	no	4425	63.8	2437	62.5	1988	65.5
Region	North/rest of UK	1980	28.5	1134	29.1	846	27.9
	midlands	1507	21.7	835	21.4	672	22.1
	south and east	3449	49.7	1931	49.5	1518	50
Education	less than GCSE/equivalent/foreign	2790	40.2	1683	43.2	1107	36.5
	GCSE/A-level/equivalent	1960	28.3	1198	30.7	762	25.1
	higher than A-level	2186	31.5	1019	26.1	1167	38.4
Whether difficult walking ¼ mile unaided	no difficulty	4890	70.5	2631	67.4	2259	74.4
	some difficulty	854	12.3	536	13.7	318	10.5
	much difficulty	425	6.1	250	6.4	175	5.8
	unable to	767	11.1	483	12.4	284	9.4

All counts are pooled means to 0 decimal places

^a^ 55 respondents were coded as ‘no’ for ‘whether a member of any organisations, clubs, or societies’, but coded elsewhere in the dataset as being a member of an organisation, club, or society. These respondents were removed (*N* = 6881)

Women were lonelier than men according to all three measures. Men’s mean UCLA score was 4.05 (SE 0.03), and women’s was 4.23 (SE 0.03). The 95% CI of the mean difference was 0.11–0.25 (*P* < 0.001). This translates to a 4.2% higher percentage of women recording a score of lonely according to the dichotomised scale (21.8% of women, 17.6% of men). The direct question showed a larger difference. 74.8% of men said they were ‘rarely/never’ lonely, compared to just 65.0% of women. Furthermore, 26.7% of women were ‘sometimes’ lonely, and 8.3% ‘often’ lonely, thus 35% of women were sometimes or often lonely (13.2% more women than a score of lonely on the UCLA scale). Conversely, 19.9% of men were ‘sometimes’ lonely, and 5.3% ‘often’, totalling just 25.2% of men (7.6% more men than those who had a score of lonely UCLA scale). In the question about loneliness in the past week, 14% of women had felt lonely, and 10% of men. Though women appeared lonelier, men displayed worse social relationships. 1.4% of men were severely socially isolated (disregarding partners/spouses), compared to 0.5% of women, and 2.3% of men had no close relationships (disregarding partners/spouses), compared to 0.8% of women. Women also averaged more close relationships (7.77 compared to 7.11) and a better view of their friendships (mean PFR 24.27 compared to 22.90).

### Regression models

Tables 4, 5 and 6 display the odds ratios (OR)/Incidence Rate Ratios (IRR) and 95% confidence intervals (CI) of the variables of interest against a single reference category. The full models, and the size and significance of the interaction terms, are in additional files 4, 5, 6, 7, 8, 9, 10, 11, 12, 13, 14 and 15. Evidence was found to support the hypothesis that men are less likely to state they are lonely on to a direct question (hypothesis 1). Table 4 shows that, even when controlling for UCLA score, men displayed significantly lower odds of stating they were sometimes or often lonely (Model 1: OR 0.72, 95% CI 0.62–0.84). The findings on whether men are more likely to drink alcohol when lonely (hypothesis 2) were mixed. Offering support for the hypothesis, men who stated they were lonely in the past week consumed the most alcohol (Table 4, model 2.2: IRR 2.62, 95% CI 2.21–3.11), whereas women who stated they were lonely drank the *least* (Table 4, model 2.2: IRR 0.83, 95% CI 0.73–0.95). However, in the multinomial regression examining frequency of days in which alcohol was consumed (model 2.1), men appeared to drink alcohol more often (men’s baseline for drinking almost every day or more OR 4.22, 95% CI 2.55–6.97, additional file 6), but UCLA score did not show much impact on either men’s or women’s alcohol consumption (Table 4), and additional file 6 shows the value of the interaction term was not statistically significant across the multinomial models (estimates of the interaction coefficient for sex by UCLA score (B) on the logit scale, ref = never- once every couple of months: once a month—twice a week B 0.029, *P*-value 0.517; three—six days a week B 0.042, *P*-value 0.459; almost every day or more B -0.079, *P*-value 0.172).

**Table 4:** Odds/incidence rate ratios of loneliness/alcohol consumption by sex

Model 1. Ordinal regression (Proportional odds). Dependent variable: how often the respondent feels lonely (rarely/never – sometimes – often)		
Independent Variables	OR	95% CI
Male (ref = female)	0.72	0.62 – 0.84
3-item UCLA score	3.97	3.73 – 4.21
Model 2.1. Multinomial regression. Dependent variable: How often consumed alcohol in last 12 months (ref = never—once every couple of months)		
Independent Variables	OR	95% CI
Odds of drinking alcohol once a month—twice a week^a^		
Women’s 3-item UCLA score	0.95	0.90 – 1.00^b^
Men’s 3-item UCLA score	0.98	0.91 – 1.05
Odds of drinking alcohol three—six days a week^c^		
Women’s 3-item UCLA score	0.90	0.83 – 0.97
Men’s 3-item UCLA score	0.94	0.86 – 1.02
Odds of drinking alcohol almost every day or more^d^		
Women’s 3-item UCLA score	0.99	0.92 – 1.08
Men’s 3-item UCLA score	0.92	0.85 – 1.01
Model 2.2. Negative binomial regression. Dependent variable: Estimated units alcohol consumed in past week (ref = women who have not felt lonely in past week)		
Independent Variables	IRR	95% CI
Women who have felt lonely in past week	0.83	0.73 – 0.95
Men who have not felt lonely in past week	2.31	2.18 – 2.47
Men who have felt lonely in past week	2.62	2.21 – 3.11

*OR* Odds Ratio, *CI* Confidence Interval. All models used pooled estimates. Covariates: ethnicity, age, employment status, health status, whether has a limiting long-standing illness, whether difficult walking 1/4 mile unaided, wealth, income, region, education, partner status, sex

^a^ Log(odds(outcome)) = constant + .491 sex + -.051 UCLA + .029 sex*UCLA + covariates. The reference group for sex was women

^b^ 1.004 at 3 decimal places

^c^Log(odds(outcome)) = constant + .770 sex + -.106 UCLA + .042 sex*UCLA + covariates

^d^Log(odds(outcome)) = constant + 1.440 sex + -.001 UCLA + -.079 sex *UCLA + covariates

**Table 5:** Impact of social relationships on loneliness in men and women using logistic regression

Dependent variable: dichotomised UCLA score (ref = a score of not lonely)		
Independent Variables	OR	95% CI
*Model 3.1. Indicator of severe isolation, excluding spouses/partners (ISI). Ref* = *Not severely isolated women*		
Not severely isolated men	1.02	0.89—01.18
Severely isolated women	7.58	2.45—23.48
Severely isolated men	1.01	0.42—02.40
*N* = 6881^a^		
*Model 3.2. Indicator of any close relationships, excluding spouses/partners (IAC). Ref* = *Women with at least one close relationship*		
Men with a close relationship	1.01	0.88—1.17
Women with no close relationships	3.40	1.50—7.71
Men with no close relationships	1.10	0.57—2.11
*N* = 6936		
*Model 3.3. Index of Close Relationships, excluding spouses/partners (ICR)*^*b*^		
Women’s number of close relationships	0.89	0.87—0.92
Men’s number of close relationships	0.91	0.89—0.94
*N* = 6936		
*Model 3.4. Perception of Friendship Relationships (PFR) score*^*c*^		
Women’s perception of friendships score	0.84	0.81—0.86
Men’s perception of friendships score	0.85	0.82—0.89
*N* = 6453.64^d^		

*OR* Odds Ratio, *CI* Confidence Interval. All models used pooled estimates. Covariates: ethnicity, age, employment status, health status, whether has a limiting long-standing illness, whether difficult walking 1/4 mile unaided, wealth, income, region, education, partner status

^a^ 55 respondents were removed as they were coded as ‘no’ for ‘whether a member of any organisations, clubs, or societies’, but coded elsewhere as being a member of an organisation, club, or society. Models including these responses, unaltered from original data, were not notably different

^b^ log(odds(outcome)) = constant -0.24 sex -0.12 ICR + 0.03 sex*ICR + covariates. The reference group for sex was women

^c^ log(odds(outcome)) = constant -0.71 sex + -0.18 PFR + 0.02 sex*PFR + covariates

^d^ Whether the respondent had any friends required imputation, therefore N is a pooled mean

**Table 6:** Impact of partner status on loneliness in men and women using logistic regression

Dependent variable: dichotomised UCLA score (ref = a score of not lonely)										
*Model investigating the impact of partner status in men and women*			*Models investigating whether social relationships mediate the association between partner status and sex*							
	Model 4		Model 5.1		Model 5.2		Model 5.3		Model 5.4	
	OR	95% CI	OR	95% CI	OR	95% CI	OR	95% CI	OR	95% CI
Partner status (ref = women in a cohabiting relationship)										
- Men in a cohabiting relationship	0.83	0.68—1.00^a^	0.82	0.68—0.99	0.82	0.68—0.99	0.78	0.64—0.94	0.63	0.51—0.77
- Not cohabiting never married women	2.63	1.82—3.67	2.52	1.74—3.66	2.58	1.79—3.72	2.27	1.56—3.29	2.45	1.64—3.67
- Not cohabiting previously married women	2.50	2.07—3.07	2.53	2.07—3.09	2.53	2.07—3.08	2.70	2.21—3.30	2.84	2.29—3.52
- Not cohabiting never married men	2.28	1.60—3.23	2.22	1.55—3.17	2.21	1.55—3.14	1.80	1.25—2.58	1.93	1.30—2.85
- Not cohabiting previously married men	3.44	2.71—4.38	3.50	2.76—4.46	3.42	2.69—4.35	3.45	2.70—4.41	2.87	2.21—3.74
Severely isolated (ISI) ^b^	-	-	2.11	1.12—3.97	-	-	-	-	-	-
No close relationships (IAC) ^b^	-	-	-	-	1.61	0.98—2.66	-	-	-	-
Number of close relationships (ICR) ^b^	-	-	-	-	-	-	0.90	0.88—0.92	-	-
Perception of friendships score (PFR)	-	-	-	-	-	-	-	-	0.84	0.82—0.86
N	6936		6881^c^		6936		6936		6453.64^d^	

*OR* Odds Ratio, *CI* Confidence Interval. All models using pooled estimates. Control variables: ethnicity, age, employment status, health status, whether has a limiting long—standing illness, whether difficult walking 1/4 mile unaided, wealth, income, region, education

^a^ 0.995 at 3dp

^b^ Excluding spouses/partners

^c^ 55 respondents were removed as they were coded as ‘no’ for ‘whether a member of any organisations, clubs, or societies’, but coded elsewhere as being a member of an organisation, club, or society. Models including these responses, unaltered from original data, were not notably different

^d^ Mean N of each imputation. Imputed N varies as the number of people who have any friends varies across each imputation model. Recomputing model 4 excluding those with no friends in model 5.4 displayed similar results to model 4 including all participants (see additional file 15)

Contrary to the hypothesis that men would be more greatly impacted by their social relationships (hypothesis 3), Table 5 shows that severely isolated men (ISI), and men with no close relationships (IAC), were no more likely to record a score of lonely than other men (model 3.1: severely isolated men OR 1.01, 95% CI 0.42–2.40; model 3.2: men with no close relationships OR 1.10, 95% CI 0.57–2.11). Women, though, showed much greater odds of a score of lonely if they were severely isolated (model 3.1: OR 7.58, 95% CI 2.45–23.48) or indicated they had no close relationships (model 3.2: OR 3.40, 95% CI 1.50–7.71). Additional files 7 and 8 show the interaction term for ISI and sex was significant with a *P*-value < 0.001 (B: -2.041), and the interaction term for IAC and sex was significant with a *P*-value of 0.034 (B: -1.141). However, for the non-dichotomised version of the IAC (ICR), the estimates for men and women show they are similarly protected by a greater total number of close relationships (model 3.3: women’s ICR OR 0.89, 95%CI 0.87–0.92; men’s ICR OR 0.91, 95% CI 0.89–0.94). The interaction term was very small and not statistically significant (B: 0.027, P-value: 0.178, additional file 9). Similarly, in model 3.4, women’s PFR score showed an OR of 0.84 (95% CI 0.81–0.86), and men’s an OR of 0.85 (95% CI 0.82–0.89), with a coefficient for the interaction term of just B: 0.019 (P-value: 0.443). This indicates men and women are similarly protected from loneliness by friendship quality.

Model 4 (Table 6) indicates support for the notion that men are more impacted by partner status than women (hypothesis 4), albeit with an important caveat. As hypothesised, previously married men who did not cohabit with a partner were the most likely sex by partner status group to record a score of lonely (OR 3.44, 95% CI 2.71—4.38), and additional file 11 shows the interaction term was statistically significant with *P*-value < 0.001 (B: 0.504). Further in keeping with the hypothesis, men who cohabited with a partner were the least lonely sex by partner status category (OR 0.83, 95% CI 0.68–1.00), although the upper confidence limit was close to 1 (0.995 at 3 decimal places). However, the coefficient for the interaction term for sex and never married and not cohabiting was not statistically significant (B: 0.048, P-value: 0.849). As with cohabiting people, then, never married and not cohabiting men were less likely to record a score of lonely than their female counterparts (not cohabiting never married men OR 2.28, 95% CI 1.82—3.67; not cohabiting never married women OR 2.63, 95% CI 1.60–3.23).

Support was found for hypothesis 5, that social relationships mediate men’s greater association between partner status and loneliness, but only for perceptions of friendships. Table 6 shows the odds of a score of lonely for men in all partner status categories were lower than in model 4 when including PFR as an explanatory variable (model 5.4). As such, the interaction term for sex and previously married but not cohabiting remained statistically significant (B: 0.477, P-value 0.004, additional file 15). Nevertheless, Table 6 shows previously married but not cohabiting men had almost identical odds of loneliness to women in the same position (men OR 2.87, 95% CI 2.21—3.74; women 2.84, 95% CI 2.29—3.52), but cohabiting men had much lower odds of a score of lonely than cohabiting women (OR 0.63, 95% CI 0.51—0.77). This indicates that PFR score mediates older men’s greater loneliness among the previously married, but also increases the benefit of cohabitation. This model could not include people with no friends, therefore model 4 was rerun with the same sample as model 5.4. This showed similar results to the original version of model 4 (additional file 15), suggesting that PFR score does indeed impact the moderating effect of sex on partner status in the prediction of loneliness. Models including ISI, IAC, and ICR as an independent variable (models 5.1, 5.2, and 5.3) showed very similar results to model 4, suggesting social isolation and the number of close relationships do not mediate older men’s greater association between partner status and loneliness.

## Discussion

The current study interrogated five hypotheses denoting sex differences in loneliness in a large England-based dataset of people aged 50 + . Men showed lower odds than women of stating they are lonely in response to a direct question even when controlling for an indirect scale of loneliness (UCLA scale). Men also displayed a greater association between alcohol consumption and loneliness when examining the previous week, but not when using a less precise measure of the past year. Men’s and women’s odds of a score of lonely were similarly impacted by social relationships in linear models, but dichotomised variables suggested men who are severely isolated/have no close relationships (disregarding spouses/partners) are *much* less affected by this than women. Previously married men showed greater odds of a score of lonely than previously married women, but cohabiting and never married men showed lower odds than their female counterparts. Perceptions of friendships mediated this association, but social isolation and number of close relationships did not.

Though a reluctance to admit mental health problems is a commonly identified and discussed aspect of research and practice on men’s mental health [7], it had not been empirically evidenced in regard to loneliness on a generalisable scale in England. The novel findings in the current study add weight to literature positing that men ‘understate’ loneliness on a direct question [14, 15]. This is consistent with theoretical literature suggesting normative ideals of masculinity eschew indications of vulnerability [67, 68]. Ratcliffe et al. [11] suggest older UK men construct loneliness as a lack of ‘social worth’. If the direct question is perceived to undermine a sense of worth more readily than the UCLA scale, this may explain why men understate loneliness even on a confidential survey. This suggests a more accurate indication of loneliness in older men can be attained by the UCLA scale than a direct question.

The two models investigating loneliness and alcohol consumption did not cohere. It may be that measuring the past week and using a direct question on loneliness (model 2.2) facilitated unrepresentative results. However, given the evidence of a common cultural association between alcohol and loneliness in men [19–21], it is theoretically more likely that measuring the number of units of alcohol consumed, and providing a clearer timeframe for identifying simultaneous loneliness and alcohol consumption (model 2.2), was able to pick up on associations the model based on the past year could not (model 2.1). Additionally, in model 2.2, women showed a significant reduction in loneliness with greater alcohol consumption. This suggests that research emphasising the benefits of social contact that co-occur with alcohol consumption [24] may be applicable to older England-based women, but not men. The descriptive statistics showed older men had poorer perceptions of their social relationships according to the ICR, IAC, and PFR. This may explain why they are not able to benefit from the increased social contact alcohol use facilitates. Alternatively, men’s higher levels of alcohol use could indicate that loneliness is associated with much more problematic use, hence a greater association with negative emotional effects.

No evidence was found to support the hypothesis that men’s loneliness is more impacted by their social relationships than women’s. When examining linear models (3.3 and 3.4), men and women showed similarly lower odds of a score of lonely as the scores improved. In models using dichotomous variables, strong evidence for the alternative hypothesis was found, such that men were much less affected by being severely isolated or having no close relationships (models 3.1 and 3.2). This could suggest that dichotomising the data led to unreliable findings. Even when comparing the ICR and IAC (the IAC is a dichotomised version of the ICR), there is only evidence of a sex difference when using the IAC. However, dichotomised variables are arguably more meaningful, as it is theoretically plausible to expect a greater difference between people with zero and some close relationships than it would be between people with, say, four and five close relationships, something the ICR considers equal to zero and one. Furthermore, the descriptive statistics indicated that men were less lonely, but that they also had poorer social relationships (Table 2 and 3). This signifies there are some men in the data who have poorer social relationships but report less loneliness. The current study therefore suggests that most older men’s and women’s UCLA scores are similarly impacted by social relationships, but that among those with very poor social relationships, women are much more impacted.

Older men’s unadjusted mean UCLA score was lower than women’s (mean difference 0.18, 95% CI 0.11–0.25). Some research has found the greater incidence of widowhood may account for greater loneliness in older women when using the direct question [12]. However, the models presented in Table 5 suggest this difference is disproportionately between severely isolated men and women. This could suggest men are more likely to be content with an isolated existence. This chimes well with the notion that loneliness is a subjective perspective, and social isolation an objective state [1, 2]. Indeed, in a qualitative study of older men’s social networks, Davidson [29] constructed the theme ‘alone, not lonely’ to encapsulate a male lack of loneliness in response to isolation.

However, regression models 3.1 – 3.4 control for partner status. Moreover, if men are less inclined to state they are lonely on a direct question as it indicates vulnerability [14, 15], it is theoretically plausible that the UCLA items can also indicate vulnerability. As well as the notion of ‘alone, not lonely’, Davidson ([29], p39) concluded that socially isolated older men constructed a ‘separateness’ from other people. The results of the current study, then, could represent severely isolated older men’s tendency to construct a notion of ‘separateness’ that can influence the odds of a score of lonely even on the UCLA scale. Research indicating social isolation is as harmful as loneliness, possibly more [47], and that living alone is a greater risk-factor for mortality in older men than older women [69], suggests that severely isolated men are likely to suffer negative effects despite a score of not lonely. Furthermore, research finding older men are more likely to be socially isolated than older women [70] emphasises the need for addressing this problem in a gender-aware manner.

The present findings add to literature indicating partner status has a greater impact on loneliness in older men than in older women [25–27]. They also add an important caveat—older men who had never married, and did not cohabit with a partner, were slightly less likely to record a score of lonely than older women in the same situation (model 4). This suggests that older men who have never married do not share other men’s dependency on spouses/partners. It was also hypothesised that poor social relationships explain men’s greater reliance on spouses/partners for preventing and alleviating loneliness. Though men were more isolated/had worse social relationships on all four measures (ISI, IAC, ICR, PFR), only PFR score reduced the difference in loneliness between previously married men and women, and it also increased the benefit to cohabitation among men (model 5.4).

This still constitutes evidence that previously married men’s greater loneliness is related to poorer friendships—it is only that better friendships may allow men to benefit even more greatly from cohabitation too. Social isolation, and close relationships within the family, may have less of an influence as these do not denote ‘belongingness’ in a public arena, which Franklin et al. [4] argue is vital to masculine constructions of loneliness. This is theoretically consistent with Chalise et al.’s [71] research in Nepal, that found older men’s likeliness of loneliness was significantly reduced by providing social support to friends and neighbours, but older women’s was not. Future research may benefit from examining why older men have a worse perception of their friendships than older women, and what can be done to improve the quality of older men’s friendships.

### Implications

These findings indicate a need for sex-sensitive policy and practice in loneliness on a national scale, such as in the UK government’s 2021 action plan [72]. Using the UCLA scale, rather than a direct question, should be recommended as more likely to provide an accurate picture of older men’s loneliness. However, it should be noted that this can still underestimate loneliness in some older men, particularly those who are severely isolated. Ideally, a measure of social isolation should also be included whenever measuring loneliness. Policy and practice should recognise the need to identify and provide support for severely isolated older men even if they do not state they are lonely. Older men who drink heavily may also be particularly in need of support for loneliness. Men’s sheds, a form of intervention where men communally engage in supported DIY activities, have been noted for their ability to successfully engage older men and improve health behaviours [73].

Older men displayed a greater association between partner status and loneliness, such that cohabiting men were the least lonely group, and previously married but not cohabiting the loneliest. It could be said that this indicates policy should aim to build, maintain, and replace such relationships when necessary. Indeed, the UK government’s 2018 loneliness strategy [74] recommends a ‘family and relationships test’ be conducted on all policy. However, whilst a national focus on promoting social relationships may be beneficial, this implies policy should influence people’s, mainly women’s, decisions on whether to marry and stay married, therefore is ethically problematic. As good friendships mediated men’s greater association between partner status and loneliness, facilitating opportunities for better quality friendships would be a more suitable goal for policy and practice. Support services for recently separated, divorced, or widowed men would also target an in-need group in a less ethically debatable manner.

### Strengths and limitations

The ISI, IAC, ICR, and PFR were not designed specifically to measure the social relationships masculinity theorists have argued men lack, rendering them imperfect measures for the analysis. Ethnicity was coded as ‘white’ or ‘non-white’, in which non-white people made up just 3% of the sample, and foreign qualifications were classed as the lowest level of educational attainment regardless of their actual level. The findings are therefore less generalisable to non-white English populations. Despite the large dataset, complete with imputations, some categories remained small, reducing the chances of a statistically significant result [75]. The data was collected prior to the Covid-19 pandemic. Social changes during that time may render aspects of the data obsolete.

Further examination of the variable measuring loneliness in the last week, and the separate items of the UCLA scale, may provide more information on sex differences in different survey items. Conducting validity and reliability tests on the loneliness measures, and the measures related to social relationships, in a UK context, would also be beneficial to future research. Though using the dichotomised UCLA scale overcame problems related to the range and skew of the data [47, 48], it still facilitates an arbitrary loss of detail, in which the difference between a score of 5 and 6 is artificially inflated [41]. Sensitivity analyses on people that had a ‘proxy’ interview may also reveal different trends.

Path analysis of the impact of the ISI, IAC, ICR, and PFR on sex differences in loneliness and partner status may provide further insight into mediatory effects. The impact of loneliness on other mental health measures included in the CES-D, and whether there are sex differences in these, may also provide useful information that was not investigated in this study. Additional files 4, 5, 6, 7, 8, 9, 10, 11, 12, 13, 14 and 15 show numerous significant results for control variables, indicating that further research into these variables, including their intersectional impact on sex differences in loneliness, may further elucidate our understanding of loneliness in older populations.

Though some variables employed were imperfect, they were the best available, and could be investigated in a single, large, well-designed dataset. Regardless of the outcome of a sensitivity analysis, proxy interviews are unsuitable for a study so focused on subjective perspectives, and particularly unsuitable for investigating the likeliness of admitting loneliness. Though reliability and validity testing may be useful, this study provides a theoretically informed investigation and interpretation of survey data, able to frame discussions of validity and reliability. Overall, the current study provided intricate, extensive, and generalisable details on sex and loneliness in England-based over 50s, adding weight, detail, and new perspectives to what is currently known about sex differences in older adults’ loneliness.

## Conclusions

A large England-based dataset was used to examine sex differences in what is associated with loneliness in older men and women. This provided empirical evidence that older men are less likely to state they are lonely than older women in response to a direct question, evidencing the theoretical notion that a masculine disinclination to display vulnerability is a barrier to alleviating loneliness in older men. Older men who are lonely were found to drink much more than other men, whereas lonely older women drink less than other women, indicating older men may be seeking solace through alcohol, whilst older women benefit from the social contact associated with alcohol use. The study extended the evidence base indicating that men are more reliant on partners/spouses, showed that men who have never married and do not cohabit may not share this trend, and provided generalisable evidence that men’s poorer friendships may explain this reliance. It found that severely isolated older men show no increase in the risk of loneliness on the UCLA scale, whereas severely isolated older women show a severe risk of loneliness, suggesting that severely isolated older men are handling and constructing their isolation markedly different to older women. These findings provide generalisable evidence for theoretical notions identified in qualitative work, and replicate findings from other cultural contexts. They are not deterministically applicable to all older men and women, and do not represent sex differences that are unchangeable across time and context. Nevertheless, they can be used as a guide for gender-sensitive policy and practice on a national scale in England, and internationally if local context is properly considered and contrasted against this data.

### Supplementary Information

**Additional file 1.** Univariate missing data statistics.

**Additional file 2.** Do file for Multiple Imputation.

**Additional file 3.** Diagnostic information and adaptations.

**Additional file 4.** Regression model 1.

**Additional file 5.** Regression model 2.1.

**Additional file 6.** Regression model 2.2.

**Additional file 7.** Regression model 3.1.

**Additional file 8.** Regression model 3.2.

**Additional file 9.** Regression model 3.3.

**Additional file 10.** Regression model 3.4.

**Additional file 11.** Regression model 4.

**Additional file 12.** Regression model 5.1.

**Additional file 13.** Regression model 5.2.

**Additional file 14.** Regression model 5.3.

**Additional file 15.** Regression model 5.4 (and remodelling of model 4).

## Data Availability

The dataset analysed during the current study (ELSA, wave 8) is available from the the UK data service repository, https://beta.ukdataservice.ac.uk/datacatalogue/series/series?id=200011.

## Abbreviations

CI: Confidence Interval
ELSA: English Longitudinal Study of Ageing
IAC: Indicator of Any Close relationships
ICR: Index of Close Relationships
IRR: Incidence Rate Ratio
ISI: Indicator of Severe Isolation
MCAR: Missing Completely at Random
MICE: Multiple imputation with Chained Equations
OR: Odds Ratio
PFR: Perception of Friendship Relationships
SE: Standard Error
UCLA: University of California, Los Angeles (loneliness scale)
UK: United Kingdom
