# Supplementary material for: Older men and loneliness: a cross-sectional study of sex differences in the English Longitudinal Study of Ageing
Source: BMC Public Health. 2024 Feb 2;24:354. doi: 10.1186/s12889-024-17892-5 (PMC10835981; doi:10.1186/s12889-024-17892-5)
Supplement: Supplementary file 1 — Additional file 1. Univariate missing data statistics. [file 12889_2024_17892_MOESM1_ESM.docx]

Additional file 1. Amount of missing data for each variable, ordered according to amount missing

| *Variables* | *Sample* | *Missing* | | |
| --- | --- | --- | --- | --- |
|  | Valid N | N | % of total sample | % of valid responses (if different) |
| How many other family members the respondent has a close relationship with | 6936 | 1452 | 20.9 | - |
| How often respondent writes to or emails children | 6936 | 1381 | 19.9 | - |
| How often the respondent writes to or emails other family members | 6936 | 1376 | 19.8 | - |
| How often the respondent writes to or emails their friends | 6936 | 1341 | 19.3 | - |
| How often the respondent sends or receives text messages from other family members | 6936 | 1180 | 17.0 | - |
| How often respondent sends or receives text messages from children | 6936 | 1114 | 16.1 | - |
| How often the respondent sends/receives text messages from friends | 6936 | 1139 | 16.4 | - |
| How many friends the respondent has a close relationship with | 6936 | 1058 | 15.3 | - |
| How many children the respondent has a close relationship with | 6936 | 1056 | 15.2 | - |
| Respondent is not a member of any organisations, clubs or societies | 6936 | 972 | 14.0 | - |
| How often respondent meets up with children on average | 6936 | 905 | 13.0 | - |
| How often respondent speaks on the phone to children | 6936 | 877 | 12.6 | - |
| How often the respondent meets up with other family members | 6936 | 848 | 12.2 | - |
| How often the respondent speaks with their friends on the phone | 6936 | 826 | 11.9 | - |
| How often the respondent speaks on the phone to other family members | 6936 | 818 | 11.8 | - |
| How much their friends criticise the respondent | 5822 | 814 | 11.7 | 14.0 |
| Number of measures of spirit the respondent had last in the last 7 days | 6936 | 798 | 11.5 | - |
| Number of pints of beer the respondent had last in the last 7 days | 6936 | 796 | 11.5 | - |
| Number of glasses of wine the respondent had last in the last 7 days | 6936 | 792 | 11.4 | - |
| How often the respondent meets up with their friends | 6936 | 784 | 11.3 | - |
| How much their friends let the respondent down | 5822 | 766 | 11.0 | 13.2 |
| Portions of vegetables (excluding potatoes) eaten on a typical day | 6936 | 758 | 10.9 | - |
| How often feels isolated from others | 6936 | 752 | 10.8 | - |
| How often their friends make too many demands on the respondent | 5822 | 744 | 10.7 | 12.8 |
| How much their friends get on the respondent's nerves | 5822 | 743 | 10.7 | 12.8 |
| How often feels left out | 6936 | 743 | 10.7 | - |
| How much respondent's friends understand the way they feel about things | 5822 | 742 | 10.7 | 12.7 |
| Portions of fruit (of any kind) eaten on a typical day | 6936 | 740 | 10.7 | - |
| How much respondent can rely on their friends if they have a serious problem | 5822 | 736 | 10.6 | 12.6 |
| How much respondent can open up to their friends if they need to talk about their worries | 5822 | 736 | 10.6 | 12.6 |
| How often had alcoholic drinks in last 12 months | 6936 | 724 | 10.4 | - |
| How often feels they lack companionship | 6936 | 717 | 10.3 | - |
| How often feels lonely | 6936 | 707 | 10.2 | - |
| Whether has a husband, wife or partner with whom they live | 6936 | 681 | 9.8 | - |
| Education | 6936 | 171 | 2.5 | - |
| BU total net (non-pension) wealth - summary var | 6936 | 90 | 1.3 | - |
| BU total net income - summary var | 6936 | 90 | 1.3 | - |
| Whether felt lonely much of the time during past week | 6936 | 35 | .5 | - |
| Whether smokes | 6936 | 23 | .3 | - |
| Marital status | 6936 | 3 | <.1 | - |
| Whether difficult walking 1/4 mile unaided | 6936 | 1 | <.1 | - |
| Whether has long-standing illness | 6936 | 1 | <.1 | - |
| Whether long-standing and limiting illness | 6936 | 1 | <.1 | - |
| (D) Definitive age variable collapsed at 90+ | 6936 | 0 | 0 | - |
| (D) Ethnicity recoded into white and non-white (consolidated) | 6936 | 0 | 0 | - |
| Best description of employment status | 6936 | 0 | 0 | - |
| Region | 6936 | 0 | 0 | - |
| Sex | 6936 | 0 | 0 | - |
