# Supplementary material for: Older men and loneliness: a cross-sectional study of sex differences in the English Longitudinal Study of Ageing
Source: BMC Public Health. 2024 Feb 2;24:354. doi: 10.1186/s12889-024-17892-5 (PMC10835981; doi:10.1186/s12889-024-17892-5)
Supplement: Supplementary file 2 — Additional file 2. Do file for Multiple Imputation. [file 12889_2024_17892_MOESM2_ESM.docx]

Additional file 2: Do file for Multiple Imputation

set maxvar 6500

mi set flong

mi register imputed scprta scprtb scprtc scprtd scprte scprtf scprtg partner heska scfeele hefunc scorg96 scako scdrpin scdrwin scdrspi scfru scveg scfrda scfrdb scfrdc scfrdd scfrde scfrdf scfrdg scfrdm scfrdh scfrdi scfrdj scfrdk scfamh scfami scfamj scfamk scfamm scchdh scchdi scchdj scchdk scchdm pscede scfeela scfeelb scfeelc totinc_bu_s nettotw_bu_s qual3 scfrd scprt heill helim

mi xeq: replace helim = 0 if heill == 0

mi xeq: replace scfrda = 0 if scfrd == 0

mi xeq: replace scfrdb = 0 if scfrd == 0

mi xeq: replace scfrdc = 0 if scfrd == 0

mi xeq: replace scfrdd = 0 if scfrd == 0

mi xeq: replace scfrde = 0 if scfrd == 0

mi xeq: replace scfrdf = 0 if scfrd == 0

mi xeq: replace scfrdg = 0 if scfrd == 0

mi xeq: replace scprta = 0 if scprt == 0

mi xeq: replace scprtb = 0 if scprt == 0

mi xeq: replace scprtc = 0 if scprt == 0

mi xeq: replace scprtd = 0 if scprt == 0

mi xeq: replace scprte = 0 if scprt == 0

mi xeq: replace scprtf = 0 if scprt == 0

mi xeq: replace scprtg = 0 if scprt == 0

mi impute chained (logit, augment) heska pscede scprt scfrd scorg96 heill (logit, augment cond (if heill==1)) helim (pmm, knn(10) cond(if scfrd==1)) scfrda scfrdb scfrdc scfrdd scfrde scfrdf scfrdg (pmm, knn(10) cond(if scprt==1)) scprta scprtb scprtc scprtd scprte scprtf scprtg (pmm, knn(10)) totinc_bu_s nettotw_bu_s scfru scveg scdrpin scdrwin scdrspi scchdm scfrdm scfamm qual3 scfeela scfeelb scfeelc scfeele scchdh scchdi scchdj scchdk scfamh scfami scfamj scfamk scfrdh scfrdi scfrdj scfrdk hefunc scako (mlogit, augment) partner = indsex indager region wpdes, add(25) rseed(873357) noisily burnin(20)
