# Supplementary material for: Older men and loneliness: a cross-sectional study of sex differences in the English Longitudinal Study of Ageing
Source: BMC Public Health. 2024 Feb 2;24:354. doi: 10.1186/s12889-024-17892-5 (PMC10835981; doi:10.1186/s12889-024-17892-5)
Supplement: Supplementary file 3 — Additional file 3. Diagnostic information and adaptations. [file 12889_2024_17892_MOESM3_ESM.docx]

**Additional file 3. Diagnostic information and adaptations.**

On all models, Variance Inflation Factors (VIF) were examined for each independent variable, and a value of above five was considered for adaptation/removal (Craney and Surles 2002). The only variables that ever did so were the interaction terms critical to the test, therefore they could not be removed. For model 1, the test of parallel lines found no imputation model, or the listwise deletion, showed a statistically significant result, suggesting the odds were proportional. The deviance statistic also suggested a good fit (p=>.999), but Pearson’s Chi^2^ was highly significant (P=<.001). A multinomial also model suggested an ordinal relationship, therefore the ordinal model was used as the final model. On all models employing the UCLA scale as the dependent variable (models 3 - 5.4), diagnostic graphs suggested there was neither a normal distribution of standardised residuals, nor uniform variance of predicted vs observed residuals. Figures A and B were taken from model 4, imputation 13, though all models and imputations saw strikingly similar results, likely due to the relatively similar nature of the models. Logistic regression, using the dichotomised UCLA score, was conducted for all models where UCLA score was the dependent variable. The Hosmer and Lemeshow test suggested these had all possessed an acceptable fit to the data. For hypothesis 5, three-way interaction between sex, partner status, and ISI/IAC/ICR/PFR were identified as potentially useful, but held cell counts too low to produce meaningful data.

For the model with the dependent variable ‘how often the respondent drunk alcohol over the past year’ (model 2.1), an ordinal regression was originally conducted. The mean test of parallel lines strongly suggested the odds were not sufficiently proportional (P=<.001). The first adaption was a multinomial logistic regression using all eight responses. As some models showed similar results, some categories were combined: ‘never’, ‘once or twice a year’, and ‘once every couple of months’ were combined to make ‘never - twice a month’; ‘once or twice a month’ and ‘once or twice a week’ became ‘once a month - twice a week’; ‘three or four days a week’ and ‘five or six days a week’ became ‘three - six days a week’, and ‘almost every day or more’ remained the highest category. Another ordinal regression examined whether the odds were sufficiently proportional using the combined four response categories, but this again showed a significance of P=<.001. The final model is a multinomial regression with the four response categories. Model 2.2, which utilised ‘whether felt lonely much in the last week’ as the dependent variable, failed to meet diagnostic criteria for Poisson regression. Poisson regression showed a Pearson/degrees of freedom value of over 7 for all imputations. Negative binomial regression, using Maximum Likelihood Estimation, was conducted.

Figure A.


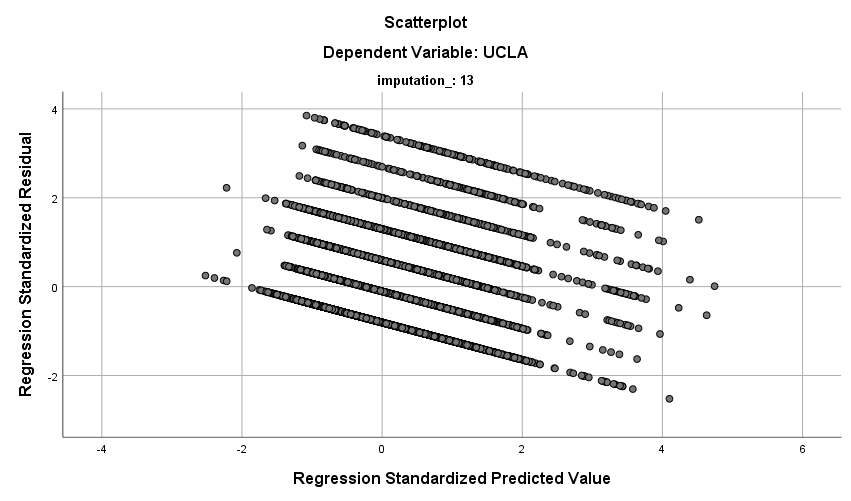


Figure B.


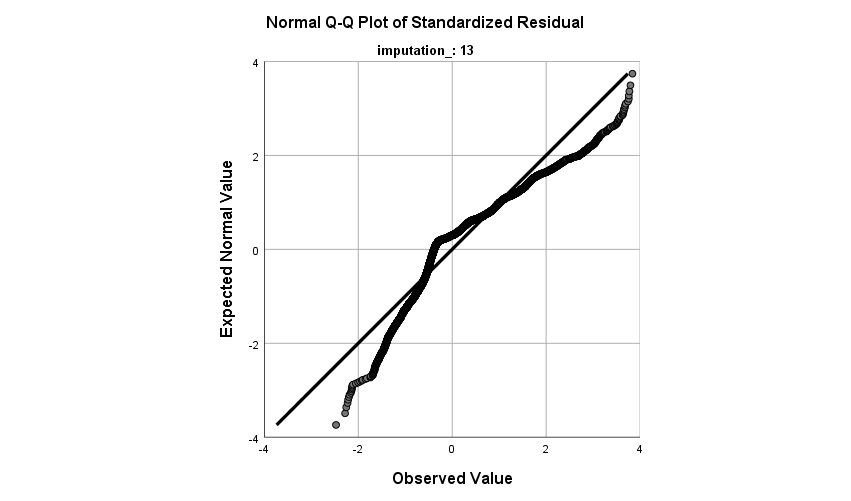


References:

Craney, T.A. and Surles, J.G., (2002). Model-dependent variance inflation factor cutoff values. Quality engineering, 14(3), 391-403.
