# Supplementary material for: Older men and loneliness: a cross-sectional study of sex differences in the English Longitudinal Study of Ageing
Source: BMC Public Health. 2024 Feb 2;24:354. doi: 10.1186/s12889-024-17892-5 (PMC10835981; doi:10.1186/s12889-024-17892-5)
Supplement: Supplementary file 4 — Additional file 4. Regression model 1. [file 12889_2024_17892_MOESM4_ESM.docx]

Additional file 4. Regression model 1.

**Ordinal regression on how often the respondent feels lonely (rarely/never>sometimes>often), using pooled estimates**

| N=6936 | B | Standard Error | P | 95% Confidence Interval | |
| --- | --- | --- | --- | --- | --- |
|  |  |  |  | lower | Upper |
| Sex (male = 1) | -.330 | .0774 | .000 | -.482 | -.178 |
| UCLA scale | 1.378 | .0307 | .000 | 1.317 | 1.438 |
|  |  |  |  |  |  |
| Ethnicity (non-white) | .236 | .1948 | .226 | -.146 | .619 |
| Age | -.009 | .0055 | .086 | -.020 | .001 |
| Partner status - first marriage and cohabiting (ref) |  |  |  |  |  |
| - Never married and not cohabiting | .382 | .1555 | .014 | .077 | .688 |
| - previously married but not cohabiting | .932 | .0897 | .000 | .756 | 1.108 |
|  |  |  |  |  |  |
| Occupation status - retired (ref) |  |  |  |  |  |
| - employed | -.135 | .1237 | .276 | -.377 | .108 |
| - Self employed | -.203 | .1878 | .279 | -.572 | .165 |
| - permanently sick/disabled | .051 | .2248 | .820 | -.390 | .493 |
| - Looking after home/family | .052 | .1786 | .771 | -.298 | .402 |
| - other | .224 | .2940 | .446 | -.352 | .801 |
| How much difficulty walking ¼ mile – none (ref) |  |  |  |  |  |
| - some | .216 | .1127 | .055 | -.005 | .437 |
| - much | .218 | .1533 | .155 | -.082 | .519 |
| - can’t | .104 | .1357 | .444 | -.162 | .370 |
| Has a limiting long-standing illness | .055 | .0898 | .538 | -.121 | .231 |
| Total wealth | -8.862E-8 | 9.1839E-8 | .337 | -2.707E-7 | 9.351E-8 |
| Total income | .000 | .0001 | .404 | .000 | .000 |
| Region – north or rest of UK |  |  |  |  |  |
| - south and east | -.039 | .0856 | .651 | -.207 | .129 |
| - midlands | .079 | .1016 | .436 | -.120 | .278 |
| Education – less than GCSE/equivalent (ref) |  |  |  |  |  |
| -GSCE/A-level/equivalent | -.121 | .0882 | .172 | -.294 | .052 |
| -Higher than A-level | -.165 | .0919 | .073 | -.345 | .015 |

**Ordinal regression on how often the respondent feels lonely (rarely/never>sometimes>often), using listwise deletion**

| N=5984 | B | Standard Error | P | 95% Confidence Interval | |
| --- | --- | --- | --- | --- | --- |
|  |  |  |  | lower | Upper |
| Sex (male = 1) | -.341 | .0813 | .000 | -.501 | -.182 |
| UCLA scale | 1.383 | .0320 | .000 | 1.321 | 1.446 |
|  |  |  |  |  |  |
| Ethnicity (non-white) | .299 | .2146 | .164 | -.122 | .719 |
| Age | -.010 | .0057 | .076 | -.021 | .001 |
| Partner status - first marriage and cohabiting (ref) |  |  |  |  |  |
| - Never married and not cohabiting | .453 | .1603 | .005 | .139 | .767 |
| - previously married but not cohabiting | .971 | .0929 | .000 | .789 | 1.153 |
|  |  |  |  |  |  |
| Occupation status - retired (ref) |  |  |  |  |  |
| - employed | -.161 | .1267 | .203 | -.410 | .087 |
| - Self employed | -.212 | .1993 | .286 | -.603 | .178 |
| - permanently sick/disabled | .003 | .2416 | .991 | -.471 | .476 |
| - Looking after home/family | .022 | .1845 | .906 | -.340 | .383 |
| - other | .120 | .3269 | .713 | -.520 | .761 |
| How much difficulty walking ¼ mile – none (ref) |  |  |  |  |  |
| - some | .161 | .1207 | .182 | -.076 | .397 |
| - much | .168 | .1626 | .301 | -.151 | .487 |
| - can’t | -.045 | .1468 | .759 | -.333 | .243 |
| Has a limiting long-standing illness | .081 | .0941 | .390 | -.104 | .265 |
| Total wealth | -1.082E-7 | 8.4149E-8 | . 199 | -2.731E-7 | 5.674E-8 |
| Total income | .000 | .0001 | .353 | .000 | .000 |
| Region – north or rest of UK |  |  |  |  |  |
| - south and east | -.018 | .0909 | .840 | -.196 | .160 |
| - midlands | .083 | .1068 | .437 | -.126 | .292 |
| Education – less than GCSE/equivalent (ref) |  |  |  |  |  |
| -GSCE/A-level/equivalent | -.104 | .0921 | .261 | -.284 | .077 |
| -Higher than A-level | -.159 | .0961 | .098 | -.347 | .030 |
