# Supplementary material for: Older men and loneliness: a cross-sectional study of sex differences in the English Longitudinal Study of Ageing
Source: BMC Public Health. 2024 Feb 2;24:354. doi: 10.1186/s12889-024-17892-5 (PMC10835981; doi:10.1186/s12889-024-17892-5)
Supplement: Supplementary file 5 — Additional file 5. Regression model 2.1. [file 12889_2024_17892_MOESM5_ESM.docx]

Additional file 5. Regression model 2.1.

**Multinomial regression on ‘How often had alcoholic drinks in last 12 months’ (ref = between never and once every couple of** **months), using pooled estimates**

| N=6936 |  | B | P | OR | 95% CI (exp)B | |
| --- | --- | --- | --- | --- | --- | --- |
|  |  |  |  |  | lower | upper |
| *Once a month - twice a week* | Intercept | -1.242 | .010 |  |  |  |
|  | Sex (male = 1) | .491 | .014 | 1.635 | 1.104 | 2.421 |
|  | UCLA (women) | -.051 | .071 | .950 | .899 | 1.004 |
|  | Interaction term for male*UCLA score | .029 | .517 | 1.029 | .943 | 1.124 |
|  | Partner status (Previously married and not cohabiting = ref) |  |  |  |  |  |
|  | Cohabiting with a partner | .464 | .003 | 1.590 | 1.177 | 2.148 |
|  | Never married and not cohabiting | .368 | .022 | 1.444 | 1.056 | 1.976 |
|  | Previously married and not cohabiting (ref) |  |  |  |  |  |
|  | Ethnicity (white) | .973 | .000 | 2.647 | 1.842 | 3.803 |
|  | Employment status (other = ref) |  |  |  |  |  |
|  | Retired | -.020 | .937 | .980 | .599 | 1.604 |
|  | Employed | -.142 | .585 | .868 | .521 | 1.445 |
|  | Self-employed | -.415 | .155 | .660 | .373 | 1.170 |
|  | Permanently sick/disabled | -.607 | .066 | .545 | .285 | 1.042 |
|  | Looking after home/family | -.305 | .287 | .737 | .421 | 1.292 |
|  | How difficult walking 1/4 mile (can’t = ref) |  |  |  |  |  |
|  | No difficulty | 1.031 | .000 | 2.804 | 2.154 | 3.649 |
|  | Some difficulty | .738 | .000 | 2.091 | 1.598 | 2.736 |
|  | Much difficulty | .395 | .016 | 1.484 | 1.078 | 2.042 |
|  |  |  |  |  |  |  |
|  | Whether has a long-standing limiting illness | .185 | .029 | 1.203 | 1.019 | 1.420 |
|  | Region (south and east = ref) |  |  |  |  |  |
|  | North | .217 | .006 | 1.242 | 1.064 | 1.449 |
|  | Midlands | .097 | .244 | 1.102 | .936 | 1.298 |
|  | Educational level (ref = higher than A - level or equivalent) |  |  |  |  |  |
|  | Less than GCSE or equivalent or foreign qualification | -.283 | .001 | .753 | .634 | .895 |
|  | GCSE or A-level or equivalent | -.128 | .158 | .880 | .737 | 1.051 |
|  |  |  |  |  |  |  |
|  | Age | -.016 | .001 | .984 | .975 | .994 |
|  | Total wealth | .000 | .000 | 1.000 | 1.000 | 1.000 |
|  | Total income | .000 | .005 | 1.000 | 1.000 | 1.001 |
| *Three - six days a week* | Intercept | -2.581 | .000 |  |  |  |
|  | Sex (male = 1) | .770 | .001 | 2.161 | 1.347 | 3.465 |
|  | UCLA (women) | -.106 | .007 | .899 | .832 | .971 |
|  | Interaction term for male*UCLA score | .042 | .459 | 1.043 | .933 | 1.166 |
|  | Partner status (Previously married and not cohabiting = ref) |  |  |  |  |  |
|  | Cohabiting with a partner | .291 | .115 | 1.338 | .931 | 1.922 |
|  | Never married and not cohabiting | .112 | .571 | 1.118 | .760 | 1.645 |
|  | Previously married and not cohabiting (ef) |  |  |  |  |  |
|  | Ethnicity (white) | 1.512 | .000 | 4.538 | 2.515 | 8.188 |
|  | Employment status (other = ref) |  |  |  |  |  |
|  | Retired | .840 | .031 | 2.316 | 1.079 | 4.970 |
|  | Employed | .611 | .121 | 1.842 | .851 | 3.988 |
|  | Self-employed | .711 | .089 | 2.035 | .897 | 4.621 |
|  | Permanently sick/disabled | .387 | .438 | 1.473 | .553 | 3.920 |
|  | Looking after home/family | .403 | .354 | 1.497 | .638 | 3.509 |
|  | How difficult walking 1/4 mile (can’t = ref) |  |  |  |  |  |
|  | No difficulty | 1.173 | .000 | 3.232 | 2.276 | 4.589 |
|  | Some difficulty | .494 | .014 | 1.639 | 1.107 | 2.427 |
|  | Much difficulty | .636 | .005 | 1.889 | 1.211 | 2.945 |
|  |  |  |  |  |  |  |
|  | Whether has a long-standing limiting illness | .304 | .003 | 1.355 | 1.109 | 1.655 |
|  | Region (south and east = ref) |  |  |  |  |  |
|  | North | .234 | .014 | 1.264 | 1.048 | 1.525 |
|  | Midlands | .000 | 1.000 | 1.000 | .818 | 1.223 |
|  | Educational level (ref = higher than A - level or equivalent) |  |  |  |  |  |
|  | Less than GCSE or equivalent or foreign qualification | -.753 | .000 | .471 | .385 | .576 |
|  | GCSE or A-level or equivalent | -.393 | .000 | .675 | .548 | .831 |
|  |  |  |  |  |  |  |
|  | Age | -.025 | .000 | .975 | .964 | .987 |
|  | Total wealth | .000 | .000 | 1.000 | 1.000 | 1.000 |
|  | Total income | .001 | .000 | 1.001 | 1.000 | 1.001 |
| *Almost every day or more* | Intercept | -4.631 | .000 |  |  |  |
|  | Sex (male = 1) | 1.440 | .000 | 4.219 | 2.554 | 6.970 |
|  | UCLA (women) | -.001 | .985 | .999 | .924 | 1.081 |
|  | Interaction term for male*UCLA score | -.079 | .172 | .924 | .825 | 1.035 |
|  | Partner status (Previously married and not cohabiting = ref) |  |  |  |  |  |
|  | Cohabiting with a partner | .185 | .355 | 1.203 | .813 | 1.780 |
|  | Never married and not cohabiting | .150 | .472 | 1.162 | .771 | 1.751 |
|  | Previously married and not cohabiting (ef) |  |  |  |  |  |
|  | Ethnicity (white) | 1.382 | .000 | 3.983 | 2.138 | 7.419 |
|  | Employment status (other = ref) |  |  |  |  |  |
|  | Retired | .722 | .102 | 2.058 | .866 | 4.894 |
|  | Employed | .513 | .255 | 1.670 | .690 | 4.044 |
|  | Self-employed | .542 | .256 | 1.719 | .675 | 4.381 |
|  | Permanently sick/disabled | .667 | .203 | 1.948 | .697 | 5.445 |
|  | Looking after home/family | .721 | .139 | 2.056 | .792 | 5.339 |
|  | How difficult walking 1/4 mile (can’t = ref) |  |  |  |  |  |
|  | No difficulty | .885 | .000 | 2.422 | 1.708 | 3.434 |
|  | Some difficulty | .408 | .035 | 1.503 | 1.030 | 2.193 |
|  | Much difficulty | .260 | .264 | 1.296 | .822 | 2.045 |
|  |  |  |  |  |  |  |
|  | Whether has a long-standing limiting illness | .222 | .048 | 1.249 | 1.002 | 1.556 |
|  | Region (south and east = ref) |  |  |  |  |  |
|  | North | .111 | .292 | 1.118 | .909 | 1.374 |
|  | Midlands | .047 | .668 | 1.049 | .844 | 1.302 |
|  | Educational level (ref = higher than A - level or equivalent) |  |  |  |  |  |
|  | Less than GCSE or equivalent or foreign qualification | -.652 | .000 | .521 | .422 | .644 |
|  | GCSE or A-level or equivalent | -.424 | .000 | .654 | .523 | .819 |
|  |  |  |  |  |  |  |
|  | Age | .000 | .957 | 1.000 | .987 | 1.012 |
|  | Total wealth | .000 | .000 | 1.000 | 1.000 | 1.000 |
|  | Total income | .001 | .000 | 1.001 | 1.000 | 1.001 |

**Multinomial regression on ‘How often had alcoholic drinks in last 12 months’ (ref = between never and once every couple of** **months), using listwise deletion**

| N=5839 |  | B | P | OR | 95% CI (exp)B | |
| --- | --- | --- | --- | --- | --- | --- |
|  |  |  |  |  | lower | upper |
| *Once a month - twice a week* | Intercept | -1.139 | .028 |  |  |  |
|  | Sex (male = 1) | .449 | .026 | 1.567 | 1.055 | 2.328 |
|  | UCLA (women) | -.052 | .064 | .949 | .898 | 1.003 |
|  | Interaction term for male*UCLA score | .039 | .392 | 1.039 | .951 | 1.136 |
|  | Partner status (Previously married and not cohabiting = ref) |  |  |  |  |  |
|  | Cohabiting with a partner | .450 | .004 | 1.568 | 1.153 | 2.133 |
|  | Never married and not cohabiting | .341 | .034 | 1.407 | 1.027 | 1.927 |
|  | Previously married and not cohabiting (ef) |  |  |  |  |  |
|  | Ethnicity (white) | 1.297 | .000 | 3.658 | 2.422 | 5.526 |
|  | Employment status (other = ref) |  |  |  |  |  |
|  | Retired | -.162 | .558 | .850 | .494 | 1.463 |
|  | Employed | -.275 | .330 | .760 | .437 | 1.320 |
|  | Self-employed | -.565 | .076 | .568 | .304 | 1.062 |
|  | Permanently sick/disabled | -.906 | .012 | .404 | .200 | .816 |
|  | Looking after home/family | -.427 | .175 | .653 | .352 | 1.209 |
|  | How difficult walking 1/4 mile (can’t = ref) |  |  |  |  |  |
|  | No difficulty | .975 | .000 | 2.651 | 2.027 | 3.468 |
|  | Some difficulty | .703 | .000 | 2.020 | 1.527 | 2.673 |
|  | Much difficulty | .450 | .010 | 1.568 | 1.115 | 2.206 |
|  |  |  |  |  |  |  |
|  | Whether has a long-standing limiting illness | .170 | .045 | 1.185 | 1.004 | 1.399 |
|  | Region (south and east = ref) |  |  |  |  |  |
|  | North | .268 | .001 | 1.308 | 1.113 | 1.537 |
|  | Midlands | .112 | .198 | 1.119 | .943 | 1.327 |
|  | Educational level (ref = higher than A - level or equivalent) |  |  |  |  |  |
|  | Less than GCSE or equivalent or foreign qualification | -.244 | .005 | .784 | .661 | .930 |
|  | GCSE or A-level or equivalent | -.084 | .362 | .920 | .768 | 1.101 |
|  |  |  |  |  |  |  |
|  | Age | -.019 | .000 | .981 | .971 | .991 |
|  | Total wealth | .000 | .000 | 1.000 | 1.000 | 1.000 |
|  | Total income | .000 | .030 | 1.000 | 1.000 | 1.001 |
| *Three - six days a week* | Intercept | -2.941 | .000 |  |  |  |
|  | Sex (male = 1) | .771 | .002 | 2.162 | 1.325 | 3.529 |
|  | UCLA (women) | -.101 | .012 | .904 | .836 | .978 |
|  | Interaction term for male*UCLA score | .029 | .617 | 1.030 | .918 | 1.155 |
|  | Partner status (Previously married and not cohabiting = ref) |  |  |  |  |  |
|  | Cohabiting with a partner | .383 | .047 | 1.466 | 1.005 | 2.138 |
|  | Never married and not cohabiting | .210 | .300 | 1.234 | .829 | 1.837 |
|  | Previously married and not cohabiting (ef) |  |  |  |  |  |
|  | Ethnicity (white) | 1.990 | .000 | 7.314 | 3.812 | 14.033 |
|  | Employment status (other = ref) |  |  |  |  |  |
|  | Retired | .705 | .091 | 2.023 | .894 | 4.581 |
|  | Employed | .510 | .227 | 1.665 | .728 | 3.806 |
|  | Self-employed | .582 | .193 | 1.790 | .745 | 4.299 |
|  | Permanently sick/disabled | .213 | .685 | 1.238 | .442 | 3.468 |
|  | Looking after home/family | .257 | .582 | 1.293 | .518 | 3.228 |
|  | How difficult walking 1/4 mile (can’t = ref) |  |  |  |  |  |
|  | No difficulty | 1.072 | .000 | 2.921 | 2.002 | 4.263 |
|  | Some difficulty | .424 | .046 | 1.528 | 1.007 | 2.318 |
|  | Much difficulty | .757 | .002 | 2.131 | 1.333 | 3.408 |
|  |  |  |  |  |  |  |
|  | Whether has a long-standing limiting illness | .310 | .004 | 1.363 | 1.106 | 1.680 |
|  | Region (south and east = ref) |  |  |  |  |  |
|  | North | .314 | .001 | 1.368 | 1.128 | 1.661 |
|  | Midlands | .024 | .824 | 1.024 | .829 | 1.265 |
|  | Educational level (ref = higher than A - level or equivalent) |  |  |  |  |  |
|  | Less than GCSE or equivalent or foreign qualification | -.723 | .000 | .485 | .396 | .595 |
|  | GCSE or A-level or equivalent | -.351 | .001 | .704 | .573 | .866 |
|  |  |  |  |  |  |  |
|  | Age | -.026 | .000 | .974 | .962 | .987 |
|  | Total wealth | .000 | .000 | 1.000 | 1.000 | 1.000 |
|  | Total income | .001 | .000 | 1.001 | 1.000 | 1.001 |
| *Almost every day or more* | Intercept | -4.597 | .000 |  |  |  |
|  | Sex (male = 1) | 1.443 | .000 | 4.235 | 2.516 | 7.128 |
|  | UCLA (women) | .006 | .892 | 1.006 | .927 | 1.090 |
|  | Interaction term for male*UCLA score | -.087 | .149 | .917 | .814 | 1.032 |
|  | Partner status (Previously married and not cohabiting = ref) |  |  |  |  |  |
|  | Cohabiting with a partner | .147 | .466 | 1.158 | .780 | 1.720 |
|  | Never married and not cohabiting | .121 | .567 | 1.129 | .746 | 1.709 |
|  |  |  |  |  |  |  |
|  | Ethnicity (white) | 1.730 | .000 | 5.641 | 2.826 | 11.257 |
|  | Employment status (other = ref) |  |  |  |  |  |
|  | Retired | .523 | .248 | 1.687 | .695 | 4.095 |
|  | Employed | .242 | .599 | 1.274 | .516 | 3.143 |
|  | Self-employed | .363 | .456 | 1.438 | .553 | 3.739 |
|  | Permanently sick/disabled | .420 | .434 | 1.522 | .531 | 4.365 |
|  | Looking after home/family | .556 | .265 | 1.743 | .656 | 4.633 |
|  | How difficult walking 1/4 mile (can’t = ref) |  |  |  |  |  |
|  | No difficulty | .681 | .000 | 1.976 | 1.390 | 2.810 |
|  | Some difficulty | .185 | .349 | 1.203 | .817 | 1.770 |
|  | Much difficulty | .158 | .510 | 1.171 | .732 | 1.875 |
|  |  |  |  |  |  |  |
|  | Whether has a long-standing limiting illness | .209 | .067 | 1.232 | .986 | 1.540 |
|  | Region (south and east = ref) | 0^b^ | . | . | . | . |
|  | North | .152 | .163 | 1.164 | .940 | 1.441 |
|  | Midlands | .034 | .767 | 1.035 | .825 | 1.297 |
|  | Educational level (ref = higher than A - level or equivalent) | 0^b^ | . | . | . | . |
|  | Less than GCSE or equivalent or foreign qualification | -.584 | .000 | .558 | .449 | .692 |
|  | GCSE or A-level or equivalent | -.400 | .001 | .670 | .533 | .843 |
|  |  |  |  |  |  |  |
|  | Age | .001 | .933 | 1.001 | .987 | 1.014 |
|  | Total wealth | .000 | .000 | 1.000 | 1.000 | 1.000 |
|  | Total income | .001 | .000 | 1.001 | 1.000 | 1.001 |
