# Supplementary material for: Older men and loneliness: a cross-sectional study of sex differences in the English Longitudinal Study of Ageing
Source: BMC Public Health. 2024 Feb 2;24:354. doi: 10.1186/s12889-024-17892-5 (PMC10835981; doi:10.1186/s12889-024-17892-5)
Supplement: Supplementary file 6 — Additional file 6. Regression model 2.2. [file 12889_2024_17892_MOESM6_ESM.docx]

Additional file 6. Regression model 2.2.

**Negative Binomial regression on alcohol consumed in past 7 days, using pooled estimates**

| N=6936 | **B** | **P** | **95% CI (Wald)** | |
| --- | --- | --- | --- | --- |
|  |  |  | *lower* | *upper* |
| Intercept (women who have not felt lonely in past 7 days) | 3.433 | .000 | 3.125 | 3.741 |
| women who have felt lonely in past 7 days | -.181 | .007 | -.313 | -.050 |
| men who have not felt lonely in past 7 days | .841 | .000 | .780 | .902 |
| Interaction term: men*lonely in past 7 days | .304 | .008 | .082 | .525 |
|  |  |  |  |  |
| *Partner status - in a cohabiting relationship (ref)* |  |  |  |  |
| Previously married but not cohabiting | -.030 | .687 | -.175 | .116 |
| Never married and not cohabiting | -.023 | .568 | -.100 | .055 |
|  |  |  |  |  |
| Ethnicity (non-white) | -.717 | .000 | -.932 | -.502 |
| *Occupation status - retired (ref)* |  |  |  |  |
| - employed | -.087 | .073 | -.182 | .008 |
| - Self employed | -.050 | .466 | -.184 | .084 |
| - permanently sick/disabled | .060 | .633 | -.187 | .307 |
| - Looking after home/family | -.100 | .181 | -.247 | .047 |
| - other | -.486 | .000 | -.743 | -.229 |
| *How much difficulty walking ¼ mile – none (ref)* |  |  |  |  |
| - some | -.244 | .000 | -.349 | -.139 |
| - much | -.330 | .000 | -.473 | -.186 |
| - can’t | -.464 | .000 | -.594 | -.334 |
| Has a limiting long-standing illness | -.091 | .014 | -.163 | -.019 |
| *Region – North or remainder of UK (ref)* |  |  |  |  |
| - South and East | -.087 | .017 | -.158 | -.016 |
| - Midlands | -.053 | .215 | -.136 | .031 |
| *Education – less than GCSE//foreign (ref)* |  |  |  |  |
| -GSCE/A-level/equivalent | .146 | .000 | .076 | .215 |
| -Higher than A-level | .237 | .000 | .163 | .311 |
|  |  |  |  |  |
| Age | -.024 | .000 | -.029 | -.020 |
| Total wealth | 9.962E-8 | .002 | 3.758E-8 | 1.617E-7 |
| Total income | .000 | .000 | .000 | .000 |

**Negative Binomial regression on alcohol consumed in past 7 days, using listwise deletion**

| N=5780 | **B** | **P** | **95% CI (Wald)** | |
| --- | --- | --- | --- | --- |
|  |  |  | *lower* | *upper* |
| Intercept (women who have not felt lonely in past 7 days) | 3.541 | .000 | 3.231 | 3.851 |
| women who have felt lonely in past 7 days | -.150 | .013 | -.269 | -.031 |
| men who have not felt lonely in past 7 days | .859 | .000 | .798 | .920 |
| Interaction term: men*lonely in past 7 days | .293 | .002 | .104 | .481 |
|  |  |  |  |  |
| *Partner status - in a cohabiting relationship (ref)* |  |  |  |  |
| Previously married but not cohabiting | -.053 | .423 | -.182 | .076 |
| Never married and not cohabiting | -.029 | .448 | -.103 | .045 |
|  |  |  |  |  |
| Ethnicity (non-white) | -1.032 | .000 | -1.220 | -.844 |
| *Occupation status - retired (ref)* |  |  |  |  |
| - employed | -.114 | .011 | -.203 | -.026 |
| - Self employed | -.028 | .670 | -.159 | .102 |
| - permanently sick/disabled | -.016 | .885 | -.226 | .195 |
| - Looking after home/family | -.064 | .390 | -.209 | .082 |
| - other | -.478 | .000 | -.718 | -.238 |
| *How much difficulty walking ¼ mile – none (ref)* |  |  |  |  |
| - some | -.243 | .000 | -.339 | -.147 |
| - much | -.315 | .000 | -.451 | -.179 |
| - can’t | -.352 | .000 | -.470 | -.233 |
| Has a limiting long-standing illness | -.094 | .009 | -.166 | -.023 |
| *Region – North or remainder of UK (ref)* |  |  |  |  |
| - South and East | -.110 | .001 | -.177 | -.043 |
| - Midlands | -.069 | .089 | -.148 | .010 |
| *Education – less than GCSE//foreign (ref)* |  |  |  |  |
| -GSCE/A-level/equivalent | .147 | .000 | .078 | .215 |
| -Higher than A-level | .229 | .000 | .160 | .298 |
|  |  |  |  |  |
| Age | -.026 | .000 | -.031 | -.022 |
| Total wealth | 1.373E-7 | .000 | 7.362E-8 | 2.009E-7 |
| Total income | .000 | .000 | .000 | .000 |
