# Supplementary material for: Older men and loneliness: a cross-sectional study of sex differences in the English Longitudinal Study of Ageing
Source: BMC Public Health. 2024 Feb 2;24:354. doi: 10.1186/s12889-024-17892-5 (PMC10835981; doi:10.1186/s12889-024-17892-5)
Supplement: Supplementary file 7 — Additional file 7. Regression model 3.1. [file 12889_2024_17892_MOESM7_ESM.docx]

Additional file 7. Regression model 3.1.

**Logistic regression on dichotomised UCLA scale (lonely=1), using pooled estimates**

| N=6881 | **B** | **P** | **95% CI (Wald)** | |
| --- | --- | --- | --- | --- |
|  |  |  | *lower* | *upper* |
| Intercept | -.234 | .540 | -.981 | .514 |
| *ISI*sex (ref - not severely isolated women)* |  |  |  |  |
| Sex (male = 1) | .023 | .755 | -.120 | .165 |
| ISI (severely isolated = 1) | 2.026 | .000 | .895 | 3.156 |
| Interaction term: ISI*sex | -2.041 | .000 | -3.507 | -0.591 |
|  |  |  |  |  |
| *Partner status - in a cohabiting relationship (ref)* |  |  |  |  |
| Previously married but not cohabiting | -.968 | .000 | -1.236 | -.700 |
| Never married and not cohabiting | .161 | .246 | -.111 | .433 |
|  |  |  |  |  |
| Ethnicity (non-white) | .308 | .096 | -.054 | .669 |
| *Occupation status - retired (ref)* |  |  |  |  |
| - employed | .062 | .588 | -.164 | .289 |
| - Self employed | .094 | .586 | -.244 | .432 |
| - permanently sick/disabled | 1.102 | .000 | .731 | 1.474 |
| - Looking after home/family | .413 | .014 | .084 | .742 |
| - other | -.025 | .928 | -.574 | .524 |
| *How much difficulty walking ¼ mile – none (ref)* |  |  |  |  |
| - some | .383 | .000 | .174 | .591 |
| - much | .484 | .001 | .205 | .763 |
| - can’t | .485 | .000 | .243 | .728 |
| Has a limiting long-standing illness | .228 | .007 | .064 | .392 |
| *Region – North or remainder of UK (ref)* |  |  |  |  |
| - Midlands | .074 | .439 | -.113 | .260 |
| - South and East | .031 | .696 | -.126 | .189 |
| *Education – less than GCSE//foreign (ref)* |  |  |  |  |
| -GSCE/A-level/equivalent | -.082 | .331 | -.249 | .084 |
| -Higher than A-level | -.189 | .030 | -.359 | -.018 |
|  |  |  |  |  |
| Age | -.011 | .021 | -.021 | -.002 |
| Total wealth | 6.049E-9 | .943 | -1.618E-7 | 1.739E-7 |
| Total income | .000 | .028 | -.001 | -3.140E-5 |

**Logistic regression on dichotomised UCLA scale (lonely=1), using listwise deletion**

| N=4425 | **B** | **P** | **95% CI (Wald)** | |
| --- | --- | --- | --- | --- |
|  |  |  | *lower* | *upper* |
| Intercept | -.264 | .589 | -1.219 | .692 |
| *ISI*sex (ref - not severely isolated women)* |  |  |  |  |
| Sex (male = 1) | .009 | .921 | -.165 | .182 |
| ISI (severely isolated = 1) | 2.030 | .001 | .855 | 3.204 |
| Interaction term: ISI*sex | -2.224 | .005 | -3.772 | -0.685 |
|  |  |  |  |  |
| *Partner status - in a cohabiting relationship (ref)* |  |  |  |  |
| Previously married but not cohabiting | -1.025 | .000 | -1.340 | -.709 |
| Never married and not cohabiting | .072 | .659 | -.248 | .392 |
|  |  |  |  |  |
| Ethnicity | .141 | .600 | -.387 | .669 |
| *Occupation status - retired (ref)* |  |  |  |  |
| - employed | .139 | .309 | -.129 | .408 |
| - Self employed | .102 | .625 | -.306 | .509 |
| - permanently sick/disabled | 1.473 | .000 | .984 | 1.963 |
| - Looking after home/family | .448 | .037 | .028 | .869 |
| - other | -.149 | .661 | -.817 | .518 |
| *How much difficulty walking ¼ mile – none (ref)* |  |  |  |  |
| - some | .231 | .084 | -.031 | .493 |
| - much | -.039 | .840 | -.413 | .336 |
| - can’t | .438 | .006 | .128 | .748 |
| Has a limiting long-standing illness | .231 | .027 | -.031 | .493 |
| *Region – North (ref)* |  |  |  |  |
| - Midlands | .137 | .244 | -.094 | .368 |
| - South and East | .017 | .868 | -.183 | .216 |
| *Education – less than GCSE//foreign (ref)* |  |  |  |  |
| -GSCE/A-level/equivalent | -.070 | .494 | -.271 | .131 |
| -Higher than A-level | -.157 | .136 | -.364 | .049 |
|  |  |  |  |  |
| Age | -.010 | .120 | -.023 | .003 |
| Total wealth | 1.344E-8 | .883 | -1.652E-7 | 1.921E-7 |
| Total income | .000 | .012 | -.001 | -8.676E-5 |
