# Supplementary material for: Older men and loneliness: a cross-sectional study of sex differences in the English Longitudinal Study of Ageing
Source: BMC Public Health. 2024 Feb 2;24:354. doi: 10.1186/s12889-024-17892-5 (PMC10835981; doi:10.1186/s12889-024-17892-5)
Supplement: Supplementary file 8 — Additional file 8. Regression model 3.2. [file 12889_2024_17892_MOESM8_ESM.docx]

Additional file 8. Regression model 3.2.

**Logistic regression on UCLA score (lonely=1), using pooled estimates**

| N=6936 | **B** | **P** | **95% CI (Wald)** | |
| --- | --- | --- | --- | --- |
|  |  |  | *lower* | *upper* |
| Intercept | -1.179 | .001 | -1.900 | -.457 |
| *IAC*Sex (ref: women with at least one close relationship)* |  |  |  |  |
| Sex (male = 1) | .010 | .890 | -.133 | .153 |
| IAC (no close relationships=1) | 1.225 | .003 | .406 | 2.044 |
| Interaction term: sex*IAC | -1.141 | .034 | -2.198 | -0.087 |
|  |  |  |  |  |
| *Partner status - in a cohabiting relationship (ref)* |  |  |  |  |
| Previously married but not cohabiting | .960 | .000 | .695 | 1.225 |
| Never married and not cohabiting | 1.118 | .000 | .953 | 1.282 |
|  |  |  |  |  |
| Ethnicity (non-white) | .276 | .128 | -.080 | .631 |
| *Occupation status - retired (ref)* |  |  |  |  |
| - employed | .084 | .465 | -.141 | .308 |
| - Self employed | .096 | .575 | -.241 | .434 |
| - permanently sick/disabled | 1.080 | .000 | .712 | 1.449 |
| - Looking after home/family | .435 | .009 | .109 | .762 |
| - other | -.044 | .876 | -.589 | .502 |
| *How much difficulty walking ¼ mile – none (ref)* |  |  |  |  |
| - some | .401 | .000 | .195 | .606 |
| - much | .474 | .001 | .197 | .751 |
| - can’t | .482 | .000 | .241 | .722 |
| Has a limiting long-standing illness | .234 |  | .070 | .397 |
| *Region – North or remainder of UK (ref)* |  |  |  |  |
| - South and East | .018 | .819 | -.138 | .175 |
| - midlands | .057 | .548 | -.128 | .242 |
| *Education – less than GCSE//foreign (ref)* |  |  |  |  |
| -GSCE/A-level/equivalent | -.090 | .284 | -.255 | .075 |
| -Higher than A-level | -.189 | .028 | -.358 | -.021 |
|  |  |  |  |  |
| Age | -.011 | .020 | -.021 | -.002 |
| Total wealth | 7.990E-9 | .924 | -1.589E-7 | 1.749E-7 |
| Total income | .000 | .021 | -.001 | -4.722E-5 |

**Logistic regression on UCLA score (lonely=1), using listwise deletion**

| N=4853 | **B** | **P** | **95% CI (Wald)** | |
| --- | --- | --- | --- | --- |
|  |  |  | *lower* | *upper* |
| Intercept | -1.088 | .015 | -1.964 | -.212 |
| *IAC*Sex (ref: women with at least one close relationship)* |  |  |  |  |
| Sex (male = 1) | -.026 | .761 | -.195 | .143 |
| IAC (no close relationships=1) | 1.201 | .000 | .286 | 2.116 |
| Interaction term: sex*IAC | -1.255 | .038 | -2.442 | -0.071 |
|  |  |  |  |  |
| *Partner status - in a cohabiting relationship (ref)* |  |  |  |  |
| Previously married but not cohabiting | 1.006 | .000 | .696 | 1.316 |
| Never married and not cohabiting | 1.157 | .000 | .966 | 1.349 |
|  |  |  |  |  |
| Ethnicity (non-white) | .423 | .073 | -.040 | .887 |
| *Occupation status - retired (ref)* |  |  |  |  |
| - employed | .073 | .578 | -.184 | .330 |
| - Self employed | .068 | .740 | -.331 | .466 |
| - permanently sick/disabled | 1.102 | .000 | .662 | 1.541 |
| - Looking after home/family | .454 | .017 | .080 | .828 |
| - other | -.229 | .515 | -.917 | .460 |
| *How much difficulty walking ¼ mile – none (ref)* |  |  |  |  |
| - some | .439 | .000 | .195 | .682 |
| - much | .425 | .011 | .095 | .755 |
| - can’t | .533 | .000 | .238 | .828 |
| Has a limiting long-standing illness | .200 | .047 | .003 | .396 |
| *Region – North or remainder of UK (ref)* |  |  |  |  |
| - South and East | .050 | .602 | -.137 | .236 |
| - midlands | .029 | .797 | -.192 | .250 |
| *Education – less than GCSE//foreign (ref)* |  |  |  |  |
| -GSCE/A-level/equivalent | -.237 | .159 | -.435 | -.038 |
| -Higher than A-level | -.135 | .019 | -.323 | .053 |
|  |  |  |  |  |
| Age | -.013 | .029 | -.025 | -.001 |
| Total wealth | -5.112E-8 | .565 | -2.251E-7 | 1.228E-7 |
| Total income | .000 | .055 | -.001 | 6.599E-6 |
