# Supplementary material for: Older men and loneliness: a cross-sectional study of sex differences in the English Longitudinal Study of Ageing
Source: BMC Public Health. 2024 Feb 2;24:354. doi: 10.1186/s12889-024-17892-5 (PMC10835981; doi:10.1186/s12889-024-17892-5)
Supplement: Supplementary file 9 — Additional file 9. Regression model 3.3. [file 12889_2024_17892_MOESM9_ESM.docx]

Additional file 9. Regression model 3.3.

**Logistic regression on UCLA score (lonely=1), using pooled estimates**

| N=6936 | **B** | **P** | **95% CI (Wald)** | |
| --- | --- | --- | --- | --- |
|  |  |  | *lower* | *upper* |
| Intercept | -.475 | .213 | -1.224 | .273 |
| Sex (male = 1) | -.236 | .097 | -.515 | .043 |
| ICR | -.115 | .000 | -.142 | -.088 |
| Interaction term: sex*ICR | .027 | .178 | -.012 | .065 |
|  |  |  |  |  |
| *Partner status - in a cohabiting relationship (ref)* |  |  |  |  |
| Previously married but not cohabiting | .830 | .000 | .563 | 1.097 |
| Never married and not cohabiting | 1.181 | .000 | 1.012 | 1.350 |
|  |  |  |  |  |
| Ethnicity (non-white) | .359 | .052 | -.003 | .722 |
| *Occupation status - retired (ref)* |  |  |  |  |
| - employed | .071 | .539 | -.155 | .296 |
| - Self employed | .123 | .480 | -.219 | .466 |
| - permanently sick/disabled | 1.061 | .000 | .679 | 1.442 |
| - Looking after home/family | .450 | .007 | .122 | .778 |
| - other | -.080 | .777 | -.629 | .470 |
| *How much difficulty walking ¼ mile – none (ref)* |  |  |  |  |
| - some | .399 | .000 | .190 | .608 |
| - much | .463 | .001 | .182 | .744 |
| - can’t | .492 | .000 | .246 | .738 |
| Has a limiting long-standing illness | .212 | .013 | .045 | .378 |
| *Region – North or remainder of UK (ref)* |  |  |  |  |
| - South and East | .005 | .949 | -.151 | .161 |
| - midlands | .048 | .612 | -.139 | .236 |
| *Education – less than GCSE//foreign (ref)* |  |  |  |  |
| -GSCE/A-level/equivalent | -.108 | .204 | -.275 | .059 |
| -Higher than A-level | -.218 | .013 | -.389 | -.046 |
|  |  |  |  |  |
| Age | -.010 | .052 | -.019 | 6.453E-5 |
| Total wealth | 4.204E-8 | .615 | -1.234E-7 | 2.074E-7 |
| Total income | .000 | .037 | -.001 | -1.744E-5 |

**Logistic regression on UCLA score (lonely=1), using listwise deletion**

| N=4853 | **B** | **P** | **95% CI (Wald)** | |
| --- | --- | --- | --- | --- |
|  |  |  | *lower* | *upper* |
| Intercept | -.213 | .647 | -1.124 | .699 |
| Sex (male = 1) | -.346 | .037 | -.670 | -.022 |
| ICR | -.134 | .000 | -.164 | -.104 |
| Interaction term Male*ICR | .034 | .139 | -.011 | .080 |
|  |  |  |  |  |
| *Partner status - in a cohabiting relationship (ref)* |  |  |  |  |
| Previously married but not cohabiting | .862 | .000 | .547 | 1.177 |
| Never married and not cohabiting | 1.220 | .000 | 1.024 | 1.416 |
|  |  |  |  |  |
| Ethnicity (non-white) | .519 | .032 | .043 | .994 |
| *Occupation status - retired (ref)* |  |  |  |  |
| - employed | .055 | .676 | -.204 | .315 |
| - Self employed | .092 | .655 | -.312 | .496 |
| - permanently sick/disabled | 1.096 | .000 | .641 | 1.550 |
| - Looking after home/family | .481 | .013 | .103 | .858 |
| - other | -.273 | .440 | -.967 | .421 |
| *How much difficulty walking ¼ mile – none (ref)* |  |  |  |  |
| - some | .439 | .001 | .191 | .687 |
| - much | .400 | .020 | .063 | .736 |
| - can’t | .542 | .000 | .242 | .842 |
| Has a limiting long-standing illness | .175 | .085 | -.024 | .373 |
| *Region – North or remainder of UK (ref)* |  |  |  |  |
| - South and East | .025 | .794 | -.164 | .214 |
| - midlands | .005 | .965 | -.219 | .229 |
| *Education – less than GCSE//foreign (ref)* |  |  |  |  |
| -GSCE/A-level/equivalent | -.142 | .868 | -.333 | .049 |
| -Higher than A-level | -.261 | .770 | -.462 | -.060 |
|  |  |  |  |  |
| Age | -.012 | .049 | -.024 | -5.886E-5 |
| Total wealth | -8.449E-9 | .925 | -1.834E-7 | 1.665E-7 |
| Total income | .000 | .082 | -.001 | 3.472E-5 |
