# Supplementary material for: Older men and loneliness: a cross-sectional study of sex differences in the English Longitudinal Study of Ageing
Source: BMC Public Health. 2024 Feb 2;24:354. doi: 10.1186/s12889-024-17892-5 (PMC10835981; doi:10.1186/s12889-024-17892-5)
Supplement: Supplementary file 10 — Additional file 10. Regression model 3.4. [file 12889_2024_17892_MOESM10_ESM.docx]

Additional file 10. Regression model 3.4.

**Logistic regression on dichotomised UCLA scale (lonely=1), using pooled estimates**

| N=6453.64* | **B** | **P** | **95% CI (Wald)** | |
| --- | --- | --- | --- | --- |
|  |  |  | *lower* | *upper* |
| Intercept | 2.777 | .000 | 1.731 | 3.823 |
| Sex (male =1) | -.711 | 217 | -1.841 | .418 |
| PFR | -.177 | .000 | -.208 | -.147 |
| Interaction term: sex*PFR | .019 | .443 | -.030 | .068 |
|  |  |  |  |  |
| *Partner status - in a cohabiting relationship (ref)* |  |  |  |  |
| Previously married but not cohabiting | 1.003 | .000 | .711 | 1.294 |
| Never married and not cohabiting | 1.220 | .000 | 1.039 | 1.401 |
|  |  |  |  |  |
| Ethnicity (non-white) | .217 | .254 | -.156 | .590 |
| *Occupation status - retired (ref)* |  |  |  |  |
| - employed | -.005 | .969 | -.249 | .240 |
| - Self employed | .052 | .776 | -.307 | .412 |
| - permanently sick/disabled | .988 | .000 | .574 | 1.402 |
| - Looking after home/family | .243 | .191 | -.121 | .607 |
| - other | -.286 | .396 | -.948 | .376 |
| *How much difficulty walking ¼ mile – none (ref)* |  |  |  |  |
| - some | .281 | .014 | .057 | .505 |
| - much | .396 | .009 | .099 | .694 |
| - can’t | .383 | .005 | .119 | .647 |
| Has a limiting long-standing illness | .247 | .006 | .070 | .424 |
| *Region – North or remainder of UK (ref)* |  |  |  |  |
| - south and east | .053 | .542 | -.118 | .225 |
| - midlands | .029 | .780 | -.172 | .230 |
| *Education – less than GCSE//foreign (ref)* |  |  |  |  |
| -GSCE/A-level/equivalent | -.076 | .404 | -.255 | .103 |
| -Higher than A-level | -.148 | .112 | -.330 | .035 |
|  |  |  |  |  |
| Age | -.008 | .122 | -.019 | .002 |
| Total wealth | -1.981E-8 | .816 | -1.869E-7 | 1.473E-7 |
| Total income | .000 | .075 | -.001 | 2.603E-5 |
| * mean N of each imputation. Imputed N varies as the number of people who have any friends varies across each imputation model. | | | | |

**Logistic regression on dichotomised UCLA scale (lonely=1), using listwise deletion**

| N=5316 | **B** | **P** | **95% CI (Wald)** | |
| --- | --- | --- | --- | --- |
|  |  |  | *lower* | *upper* |
| Intercept | 2.963 | .000 | 1.821 | 4.105 |
| Sex (male =1) | -.632 | .287 | -1.793 | .530 |
| *PFR*sex* |  |  |  |  |
| Women’s PFR score | -.180 | .000 | -.212 | -.147 |
| Men’s PFR score | -.165 | .000 | -.205 | -.126 |
|  |  |  |  |  |
| *Partner status - in a cohabiting relationship (ref)* |  |  |  |  |
| Previously married but not cohabiting | 1.104 | .000 | .792 | 1.416 |
| Never married and not cohabiting | 1.262 | .000 | 1.073 | 1.451 |
|  |  |  |  |  |
| Ethnicity (non-white) | .148 | .520 | -.303 | .598 |
| *Occupation status - retired (ref)* |  |  |  |  |
| - employed | .019 | .883 | -.237 | .275 |
| - Self employed | -.004 | .985 | -.404 | .397 |
| - permanently sick/disabled | 1.056 | .000 | .593 | 1.520 |
| - Looking after home/family | .319 | .097 | -.058 | .695 |
| - other | -.664 | .079 | -1.403 | .076 |
| *How much difficulty walking ¼ mile – none (ref)* |  |  |  |  |
| - some | .239 | .055 | -.005 | .483 |
| - much | .297 | .069 | -.023 | .618 |
| - can’t | .288 | .052 | -.002 | .577 |
| Has a limiting long-standing illness | .292 | .003 | .100 | .484 |
| *Region – North or remainder of UK (ref)* |  |  |  |  |
| - South and East | .024 | .799 | -.161 | .209 |
| - Midlands | -.003 | .975 | -.221 | .214 |
| *Education – less than GCSE//foreign (ref)* |  |  |  |  |
| -GSCE/A-level/equivalent | -.060 | .528 | -.247 | .127 |
| -Higher than A-level | -.126 | .206 | -.321 | .069 |
|  |  |  |  |  |
| Age | -.010 | .077 | -.022 | .001 |
| Total wealth | -1.295E-8 | .881 | -1.823E-7 | 1.565E-7 |
| Total income | .000 | .037 | -.001 | -1.893E-5 |
