# Supplementary material for: Older men and loneliness: a cross-sectional study of sex differences in the English Longitudinal Study of Ageing
Source: BMC Public Health. 2024 Feb 2;24:354. doi: 10.1186/s12889-024-17892-5 (PMC10835981; doi:10.1186/s12889-024-17892-5)
Supplement: Supplementary file 11 — Additional file 11. Regression model 4. [file 12889_2024_17892_MOESM11_ESM.docx]

Additional file 11. Regression model 4.

**Logistic regression on UCLA score (lonely=1), pooled estimates**

| N=6936 | **B** | **P** | **95% CI (Wald)** | |
| --- | --- | --- | --- | --- |
|  |  |  | *lower* | *upper* |
| Constant | -1.122 | .002 | -1.842 | -.402 |
| *Partners status*sex (ref: cohabiting women)* |  |  |  |  |
| Sex (male=1) | -.192 | .044 | -.380 | -.005 |
| Partner status - not cohabiting and never married | .966 | .000 | .600 | 1.332 |
| Partner status - not cohabiting but previously married | .924 | .000 | .726 | 1.123 |
| Interaction term: Sex*not cohabiting and never married | .048 | .849 | 0.209 | 0.800 |
| Interaction term: Sex*not cohabiting but previously married | .504 | .001 | -0.451 | 0.549 |
|  |  |  |  |  |
| Ethnicity (non-white) | .282 | .121 | -.075 | .638 |
| *Occupation status - retired (ref)* |  |  |  |  |
| - employed | .107 | .353 | -.118 | .332 |
| - Self employed | .119 | .491 | -.220 | .458 |
| - permanently sick/disabled | 1.085 | .000 | .716 | 1.454 |
| - Looking after home/family | .397 | .016 | .073 | .721 |
| - other | -.020 | .941 | -.563 | .523 |
| *How much difficulty walking ¼ mile – none (ref)* |  |  |  |  |
| - some | .402 | .000 | .197 | .607 |
| - much | .473 | .001 | .196 | .750 |
| - can’t | .485 | .000 | .244 | .726 |
| Has a limiting long-standing illness | .231 | .006 | .067 | .394 |
| *Region – North or remainder of UK (ref)* |  |  |  |  |
| - South and East | .020 | .807 | -.137 | .176 |
| - Midlands | .054 | .570 | -.132 | .239 |
| *Education – less than GCSE//foreign (ref)* |  |  |  |  |
| -GSCE/A-level/equivalent | -.092 | .273 | -.257 | .073 |
| -Higher than A-level | -.184 | .033 | -.353 | -.015 |
|  |  |  |  |  |
| Age | -.011 | .032 | -.020 | -.001 |
| Total wealth | 7.461E-9 | .931 | -1.621E-7 | 1.771E-7 |
| Total income | .000 | .014 | -.001 | -6.857E-5 |

**Logistic regression on UCLA score (lonely=1), listwise deletion**

| N=5902 | **B** | **P** | **95% CI (Wald)** | |
| --- | --- | --- | --- | --- |
|  |  |  | *lower* | *upper* |
| Constant | -1.228 | .002 | -1.998 | -.458 |
| *Partners status*sex (ref: cohabiting women)* | - | - | - | - |
| Sex (male=1) | -.206 | .040 | -.404 | -.009 |
| Partner status - not cohabiting and never married | 1.064 | .000 | .672 | 1.456 |
| Partner status - not cohabiting but previously married | .955 | .000 | .752 | 1.159 |
| Interaction term: Sex*not cohabiting and never married | -.045 | .868 | -0.579 | 0.489 |
| Interaction term: Sex*not cohabiting but previously married | .486 | .002 | 0.175 | 0.797 |
|  |  |  |  |  |
| Ethnicity (non-white) | .275 | .184 | -.130 | .680 |
| *Occupation status - retired (ref)* |  |  |  |  |
| - employed | .151 | .199 | -.080 | .383 |
| - Self employed | .090 | .629 | -.275 | .454 |
| - permanently sick/disabled | 1.189 | .000 | .786 | 1.592 |
| - Looking after home/family | .505 | .003 | .176 | .834 |
| - other | -.194 | .523 | -.787 | .400 |
| *How much difficulty walking ¼ mile – none (ref)* |  |  |  |  |
| - some | .365 | .001 | .148 | .581 |
| - much | .381 | .010 | .092 | .670 |
| - can’t | .459 | .000 | .204 | .715 |
| Has a limiting long-standing illness | .237 | .007 | .065 | .410 |
| *Region – North or remainder of UK (ref)* |  |  |  |  |
| - South and East | .032 | .708 | -.134 | .198 |
| - Midlands | .058 | .563 | -.138 | .253 |
| *Education – less than GCSE//foreign (ref)* |  |  |  |  |
| -GSCE/A-level/equivalent | -.062 | .076 | -.230 | .106 |
| -Higher than A-level | -.160 | .469 | -.337 | .017 |
|  |  |  |  |  |
| Age | -.009 | .072 | -.020 | .001 |
| Total wealth | -4.033E-8 | .617 | -1.984E-7 | 1.177E-7 |
| Total income | .000 | .016 | -.001 | -6.367E-5 |
