# Supplementary material for: Older men and loneliness: a cross-sectional study of sex differences in the English Longitudinal Study of Ageing
Source: BMC Public Health. 2024 Feb 2;24:354. doi: 10.1186/s12889-024-17892-5 (PMC10835981; doi:10.1186/s12889-024-17892-5)
Supplement: Supplementary file 12 — Additional file 12. Regression model 5.1. [file 12889_2024_17892_MOESM12_ESM.docx]

Additional file 12. Regression model 5.1.

**Logistic regression on UCLA score (lonely=1), pooled estimates**

| N=6881 | **B** | **P** | **95% CI (Wald)** | |
| --- | --- | --- | --- | --- |
|  |  |  | *lower* | *upper* |
| Constant | -1.177 | .000 | -1.900 | -.455 |
| ISI (severely isolated = 1) | .748 | .020 | .116 | 1.379 |
| *Partners status*sex (ref: cohabiting women)* |  |  |  |  |
| Sex (male=1) | -.201 | .037 | -.389 | -.013 |
| Partner status - not cohabiting and never married | .926 | .000 | .553 | 1.298 |
| Partner status - not cohabiting but previously married | .928 | .000 | .728 | 1.127 |
| Interaction term: Sex*not cohabiting and never married | .072 | .779 | -0.434 | 0.578 |
| Interaction term: Sex*not cohabiting but previously married | .528 | .001 | 0.116 | 0.824 |
|  |  |  |  |  |
| Ethnicity (non-white) | .317 | .086 | -.045 | .679 |
| *Occupation status - retired (ref)* |  |  |  |  |
| - employed | .089 | .440 | -.138 | .317 |
| - Self employed | .123 | .479 | -.217 | .462 |
| - permanently sick/disabled | 1.095 | .000 | .724 | 1.467 |
| - Looking after home/family | .380 | .022 | .054 | .707 |
| - other | -.033 | .905 | -.583 | .516 |
| *How much difficulty walking ¼ mile – none (ref)* |  |  |  |  |
| - some | .388 | .000 | .180 | .596 |
| - much | .489 | .001 | .210 | .769 |
| - can’t | .489 | .000 | .247 | .731 |
| Has a limiting long-standing illness | .220 | .009 | .056 | .385 |
| *Region – North or remainder of UK (ref)* |  |  |  |  |
| - South and East | .025 | .755 | -.133 | .183 |
| - Midlands | .067 | .483 | -.120 | .253 |
| *Education – less than GCSE//foreign (ref)* |  |  |  |  |
| -GSCE/A-level/equivalent | -.078 | .355 | -.245 | .088 |
| -Higher than A-level | -.176 | .044 | -.347 | -.005 |
|  |  |  |  |  |
| Age | -.010 | .042 | -.020 | .000 |
| Total wealth | 4.014E-9 | .963 | -1.679E-7 | 1.759E-7 |
| Total income | .000 | .020 | -.001 | -5.061E-5 |

**Logistic regression on UCLA score (lonely=1), listwise deletion**

| N=4455 | **B** | **P** | **95% CI (Wald)** | |
| --- | --- | --- | --- | --- |
|  |  |  | *lower* | *upper* |
| Constant | -1.189 | .013 | -2.123 | -.254 |
| ISI (severely isolated = 1) | .715 | .051 | -.004 | 1.434 |
| *Partners status*sex (ref: cohabiting women)* |  |  |  |  |
| Sex (male=1) | -.261 | .026 | -.490 | -.032 |
| Partner status - not cohabiting and never married | .997 | .000 | .536 | 1.457 |
| Partner status - not cohabiting but previously married | .826 | .000 | .578 | 1.073 |
| Interaction term: Sex*not cohabiting and never married | .034 | .913 | -0.571 | 0.640 |
| Interaction term: Sex*not cohabiting but previously married | .677 | .001 | 0.315 | 1.040 |
|  |  |  |  |  |
| Ethnicity (non-white) | .130 | .634 | -.404 | .663 |
| *Occupation status - retired (ref)* |  |  |  |  |
| - employed | .169 | .218 | -.100 | .437 |
| - Self employed | .132 | .526 | -.277 | .541 |
| - permanently sick/disabled | 1.429 | .000 | .939 | 1.920 |
| - Looking after home/family | .387 | .071 | -.032 | .806 |
| - other | -.139 | .682 | -.806 | .528 |
| *How much difficulty walking ¼ mile – none (ref)* |  |  |  |  |
| - some | .246 | .065 | -.016 | .508 |
| - much | -.043 | .823 | -.419 | .333 |
| - can’t | .444 | .005 | .133 | .755 |
| Has a limiting long-standing illness | .224 | .034 | .017 | .430 |
| *Region – North or remainder of UK (ref)* |  |  |  |  |
| - South and East | .006 | .956 | -.193 | .205 |
| - Midlands | .122 | .299 | -.108 | .353 |
| *Education – less than GCSE//foreign (ref)* |  |  |  |  |
| -GSCE/A-level/equivalent | -.061 | .552 | -.261 | .140 |
| -Higher than A-level | -.144 | .172 | -.351 | .063 |
|  |  |  |  |  |
| Age | -.009 | .154 | -.022 | .003 |
| Total wealth | 4.624E-9 | .959 | -1.729E-7 | 1.821E-7 |
| Total income | .000 | .008 | -.001 | .000 |
