# Supplementary material for: Older men and loneliness: a cross-sectional study of sex differences in the English Longitudinal Study of Ageing
Source: BMC Public Health. 2024 Feb 2;24:354. doi: 10.1186/s12889-024-17892-5 (PMC10835981; doi:10.1186/s12889-024-17892-5)
Supplement: Supplementary file 13 — Additional file 13. Regression model 5.2. [file 12889_2024_17892_MOESM13_ESM.docx]

Additional file 13. Regression model 5.2.

**Logistic regression on UCLA score (lonely=1), pooled estimates**

| N=6936 | **B** | **P** | **95% CI (Wald)** | |
| --- | --- | --- | --- | --- |
|  |  |  | *lower* | *upper* |
| Constant | -1.143 | .002 | -1.863 | -.423 |
| IAC (no close relationships = 1) | .479 | .059 | -.019 | .977 |
| *Partners status*sex (ref: cohabiting women)* |  |  |  |  |
| Sex (male=1) | -.200 | .037 | -.388 | -.012 |
| Partner status - not cohabiting and never married | .948 | .000 | .581 | 1.315 |
| Partner status - not cohabiting but previously married | .927 | .000 | .729 | 1.125 |
| Interaction term: Sex*not cohabiting and never married | .043 | .867 | -0.411 | 0.543 |
| Interaction term: Sex*not cohabiting but previously married | .503 | .000 | 0.207 | 0.798 |
|  |  |  |  |  |
| Ethnicity (non-white) | .287 | .114 | -.069 | .643 |
| *Occupation status - retired (ref)* |  |  |  |  |
| - employed | .108 | .346 | -.117 | .333 |
| - Self employed | .116 | .500 | -.222 | .455 |
| - permanently sick/disabled | 1.079 | .000 | .709 | 1.449 |
| - Looking after home/family | .399 | .016 | .075 | .723 |
| - other | -.050 | .858 | -.597 | .497 |
| *How much difficulty walking ¼ mile – none (ref)* |  |  |  |  |
| - some | .404 | .000 | .199 | .610 |
| - much | .472 | .001 | .194 | .750 |
| - can’t | .483 | .000 | .242 | .724 |
| Has a limiting long-standing illness | .229 | .006 | .066 | .393 |
| *Region – North or remainder of UK (ref)* |  |  |  |  |
| - South and East | .017 | .590 | -.139 | .174 |
| - Midlands | .051 | .830 | -.134 | .236 |
| *Education – less than GCSE//foreign (ref)* |  |  |  |  |
| -GSCE/A-level/equivalent | -.091 | .277 | -.257 | .074 |
| -Higher than A-level | -.182 | .035 | -.351 | -.013 |
|  |  |  |  |  |
| Age | -.010 | .037 | -.020 | -.001 |
| Total wealth | 7.791E-9 | .927 | -1.614E-7 | 1.770E-7 |
| Total income | .000 | .015 | -.001 | -6.561E-5 |

**Logistic regression on UCLA score (6+/lonely=1), listwise deletion**

| N=4853 | **B** | **P** | **95% CI (Wald)** | |
| --- | --- | --- | --- | --- |
|  |  |  | *lower* | *upper* |
| Constant | -1.019 | .022 | -1.894 | -.144 |
| IAC (no close relationships = 1) | .393 | .182 | -.183 | .969 |
| *Partners status*sex (ref: cohabiting women)* |  |  |  |  |
| Sex (male=1) | -.199 | .079 | -.422 | .023 |
| Partner status - not cohabiting and never married | 1.053 | .000 | .618 | 1.488 |
| Partner status - not cohabiting but previously married | .999 | .000 | .771 | 1.228 |
| Interaction term: Sex*not cohabiting and never married | -.059 | .843 | -0.646 | 0.528 |
| Interaction term: Sex*not cohabiting but previously married | .441 | .014 | .089 | 0.794 |
|  |  |  |  |  |
| Ethnicity (non-white) | .417 | .079 | -.048 | .883 |
| *Occupation status - retired (ref)* |  |  |  |  |
| - employed | .091 | .489 | -.166 | .347 |
| - Self employed | .083 | .682 | -.316 | .483 |
| - permanently sick/disabled | 1.093 | .000 | .652 | 1.533 |
| - Looking after home/family | .425 | .026 | .052 | .798 |
| - other | -.224 | .523 | -.912 | .463 |
| *How much difficulty walking ¼ mile – none (ref)* |  |  |  |  |
| - some | .440 | .000 | .196 | .684 |
| - much | .413 | .014 | .084 | .743 |
| - can’t | .534 | .000 | .239 | .829 |
| Has a limiting long-standing illness | .195 | .052 | -.002 | .392 |
| *Region – North or remainder of UK (ref)* |  |  |  |  |
| - South and East | .046 | .627 | -.140 | .232 |
| - Midlands | .019 | .864 | -.202 | .240 |
| *Education – less than GCSE//foreign (ref)* |  |  |  |  |
| -GSCE/A-level/equivalent | -.135 | .158 | -.323 | .053 |
| -Higher than A-level | -.233 | .022 | -.432 | -.034 |
|  |  |  |  |  |
| Age | -.013 | .034 | -.025 | -.001 |
| Total wealth | -5.349E-8 | .545 | -2.268E-7 | 1.198E-7 |
| Total income | .000 | .042 | -.001 | -1.095E-5 |
