# Supplementary material for: Older men and loneliness: a cross-sectional study of sex differences in the English Longitudinal Study of Ageing
Source: BMC Public Health. 2024 Feb 2;24:354. doi: 10.1186/s12889-024-17892-5 (PMC10835981; doi:10.1186/s12889-024-17892-5)
Supplement: Supplementary file 14 — Additional file 14. Regression model 5.3. [file 12889_2024_17892_MOESM14_ESM.docx]

Additional file 14. Regression model 5.3.

**Logistic regression on dichotomised UCLA scale (lonely=1), using pooled estimates**

| N=6936 | **B** | **P** | **95% CI (Wald)** | |
| --- | --- | --- | --- | --- |
|  |  |  | *lower* | *upper* |
| Constant | -.536 | .153 | -1.273 | .200 |
| ICR | -.104 | .000 | -.124 | -.084 |
| *Partners status*sex (ref: cohabiting women)* |  |  |  |  |
| Sex (male=1) | -.252 | .010 | -.444 | -.060 |
| Partner status - not cohabiting and never married | .818 | .000 | .446 | 1.191 |
| Partner status - not cohabiting but previously married | .993 | .000 | .791 | 1.194 |
| Interaction term: Sex*not cohabiting and never married | .020 | .940 | -0.489 | 0.528 |
| Interaction term: Sex*not cohabiting but previously married | .499 | .001 | 0.201 | 0.796 |
|  |  |  |  |  |
| Ethnicity (non-white) | .372 | .045 | .009 | .735 |
| *Occupation status - retired (ref)* |  |  |  |  |
| - employed | .094 | .415 | -.133 | .321 |
| - Self employed | .139 | .430 | -.206 | .484 |
| - permanently sick/disabled | 1.069 | .000 | .687 | 1.451 |
| - Looking after home/family | .414 | .013 | .088 | .741 |
| - other | -.082 | .772 | -.634 | .471 |
| *How much difficulty walking ¼ mile – none (ref)* |  |  |  |  |
| - some | .405 | .000 | .196 | .614 |
| - much | .465 | .001 | .183 | .746 |
| - can’t | .497 | .000 | .250 | .743 |
| Has a limiting long-standing illness | .206 | .016 | .039 | .373 |
| *Region – North or remainder of UK (ref)* |  |  |  |  |
| - South and East | .003 | .972 | -.154 | .159 |
| - Midlands | .045 | .636 | -.142 | .233 |
| *Education – less than GCSE//foreign (ref)* |  |  |  |  |
| -GSCE/A-level/equivalent | -.211 | .194 | -.383 | -.038 |
| -Higher than A-level | -.111 | .017 | -.277 | .056 |
|  |  |  |  |  |
| Age | -.008 | .090 | -.018 | .001 |
| Total wealth | 4.513E-8 | .599 | -1.247E-7 | 2.150E-7 |
| Total income | .000 | .026 | -.001 | -3.647E-5 |

**Logistic regression on dichotomised UCLA scale (lonely=1), using listwise deletion**

| N=4853 | **B** | **P** | **95% CI (Wald)** | |
| --- | --- | --- | --- | --- |
|  |  |  | *lower* | *upper* |
| Constant | -.274 | .546 | -1.178 | .623 |
| ICR | -.120 |  | -.143 | -.097 |
| *Partners status*sex (ref: cohabiting women)* |  |  |  |  |
| Sex (male=1) | -.274 | .017 | -.499 | -.049 |
| Partner status - not cohabiting and never married | .911 | .000 | .468 | 1.353 |
| Partner status - not cohabiting but previously married | 1.069 | .000 | .835 | 1.302 |
| Interaction term: Sex*not cohabiting and never married | -.098 | .748 | -0.695 | .499 |
| Interaction term: Sex*not cohabiting but previously married | .421 | .022 | 0.062 | 0.779 |
|  |  |  |  |  |
| Ethnicity (non-white) | .520 | .032 | .044 | .996 |
| *Occupation status - retired (ref)* |  |  |  |  |
| - employed | .072 | .587 | -.188 | .332 |
| - Self employed | .099 | .632 | -.307 | .505 |
| - permanently sick/disabled | 1.103 | .000 | .648 | 1.559 |
| - Looking after home/family | .453 | .018 | .076 | .829 |
| - other | -.271 | .447 | -.968 | .427 |
| *How much difficulty walking ¼ mile – none (ref)* |  |  |  |  |
| - some | .445 | .000 | .197 | .693 |
| - much | .399 | .020 | .062 | .736 |
| - can’t | .546 | .000 | .246 | .846 |
| Has a limiting long-standing illness | .166 | .101 | -.032 | .365 |
| *Region – North or remainder of UK (ref)* |  |  |  |  |
| - South and East | .021 | .829 | -.168 | .209 |
| - Midlands | .000 | .998 | -.224 | .225 |
| *Education – less than GCSE//foreign (ref)* |  |  |  |  |
| -GSCE/A-level/equivalent | -.146 | .135 | -.336 | .045 |
| -Higher than A-level | -.256 | .013 | -.458 | -.054 |
|  |  |  |  |  |
| Age | -.011 | .065 | -.023 | .001 |
| Total wealth | -8.274E-9 | .926 | -1.832E-7 | 1.667E-7 |
| Total income | .000 | .063 | -.001 | 1.631E-5 |
