# Supplementary material for: Older men and loneliness: a cross-sectional study of sex differences in the English Longitudinal Study of Ageing
Source: BMC Public Health. 2024 Feb 2;24:354. doi: 10.1186/s12889-024-17892-5 (PMC10835981; doi:10.1186/s12889-024-17892-5)
Supplement: Supplementary file 15 — Additional file 15. Regression model 5.4 (and remodelling of model 4). [file 12889_2024_17892_MOESM15_ESM.docx]

Additional file 15. Regression model 5.4 (and remodelling of model 4 excluding people with no friends to match sample in model 5.4)

**Logistic regression on dichotomised UCLA scale (lonely = 1), using pooled estimates**

| N=6453.64* | **B** | **P** | **95% CI (Wald)** | |
| --- | --- | --- | --- | --- |
|  |  |  | *lower* | *upper* |
| Constant | 2.609 | .001 | 1.666 | 3.552 |
| PFR | -.170 | .000 | -.193 | -.146 |
| *Partners status*sex (ref: cohabiting women)* |  |  |  |  |
| Sex (male=1) | -.465 | .001 | -.674 | -.255 |
| Partner status - not cohabiting and never married | .898 | .001 | .495 | 1.300 |
| Partner status - not cohabiting but previously married | 1.044 | .000 | .828 | 1.259 |
| Interaction term: Sex*not cohabiting and never married | .222 | .430 | 0.153 | 0.801 |
| Interaction term: Sex*not cohabiting but previously married | .477 | .004 | -0.330 | 0.774 |
|  |  |  |  |  |
| Ethnicity (non-white) | .230 | .229 | -.144 | .604 |
| *Occupation status - retired (ref)* |  |  |  |  |
| - employed | .016 | .900 | -.230 | .261 |
| - Self employed | .063 | .734 | -.299 | .424 |
| - permanently sick/disabled | .983 | .000 | .569 | 1.398 |
|  |  |  |  |  |
| - Looking after home/family | .214 | .245 | -.147 | .576 |
| - other | -.297 | .382 | -.964 | .370 |
| *How much difficulty walking ¼ mile – none (ref)* |  |  |  |  |
| - some | .285 | .013 | .061 | .509 |
| - much | .398 | .009 | .100 | .696 |
| - can’t | .391 | .004 | .127 | .655 |
| Has a limiting long-standing illness | .241 | .008 | .064 | .419 |
| *Region – North or remainder of UK (ref)* |  |  |  |  |
| - South and East | .055 | .529 | -.117 | .227 |
| - Midlands | .027 | .792 | -.174 | .228 |
| *Education – less than GCSE//foreign (ref)* |  |  |  |  |
| -GSCE/A-level/equivalent | -.077 | .400 | -.255 | .102 |
| -Higher than A-level | -.137 | .141 | -.320 | .046 |
|  |  |  |  |  |
| Age | -.007 | .187 | -.018 | .003 |
| Total wealth | -1.952E-8 | .818 | -1.866E-7 | 1.476E-7 |
| Total income | .000 | .058 | -.001 | 9.787E-6 |
| * mean N of each imputation. Imputed N varies as the number of people who have any friends varies across each imputation model. | | | | |

**Logistic regression on dichotomised UCLA scale (lonely=1), using listwise deletion**

| N=5316 | **B** | **P** | **95% CI (Wald)** | |
| --- | --- | --- | --- | --- |
|  |  |  | *lower* | *upper* |
| Constant | 2.849 | .000 | 1.824 | 3.875 |
| PFR | -.174 | .000 | -.199 | -.148 |
| *Partners status*sex (ref: cohabiting women)* |  |  |  |  |
| Sex (male=1) | -.482 | .039 | -.705 | -.258 |
| Partner status - not cohabiting and never married | 1.007 | .000 | .565 | 1.449 |
| Partner status - not cohabiting but previously married | 1.100 | .000 | .876 | 1.324 |
| Interaction term: Sex*not cohabiting and never married | .204 | .505 | -0.396 | 0.804 |
| Interaction term: Sex*not cohabiting but previously married | .451 | .011 | 0.104 | 0.797 |
|  |  |  |  |  |
| Ethnicity (non-white) | .153 | .507 | -.299 | .605 |
| *Occupation status - retired (ref)* |  |  |  |  |
| - employed | .039 | .767 | -.218 | .295 |
| - Self employed | .006 | .975 | -.396 | .409 |
| - permanently sick/disabled | 1.049 | .000 | .583 | 1.515 |
| - Looking after home/family | .293 | .126 | -.082 | .667 |
| - other | -.671 | .077 | -1.414 | .073 |
| *How much difficulty walking ¼ mile – none (ref)* |  |  |  |  |
| - some | .240 | .053 | -.004 | .484 |
| - much | .296 | .071 | -.026 | .617 |
| - can’t | .293 | .048 | .003 | .583 |
| Has a limiting long-standing illness | .289 | .003 | .097 | .482 |
| *Region – North or remainder of UK (ref)* |  |  |  |  |
| - South and East | .024 | .800 | -.161 | .208 |
| - Midlands | -.007 | .950 | -.224 | .211 |
| *Education – less than GCSE//foreign (ref)* |  |  |  |  |
| -GSCE/A-level/equivalent | -.059 | .534 | -.246 | .128 |
| -Higher than A-level | -.116 | .243 | -.312 | .079 |
|  |  |  |  |  |
| Age | -.010 | .107 | -.021 | .002 |
| Total wealth | -1.369E-8 | .874 | -1.828E-7 | 1.555E-7 |
| Total income | .000 | .027 | -.001 | -3.843E-5 |

**Logistic regression on dichotomised UCLA scale (lonely = 1), using pooled estimates. Excluding people with no friends.**

| N=6453.64* | **B** | **P** | **95% CI (Wald)** | |
| --- | --- | --- | --- | --- |
|  |  |  | *lower* | *upper* |
| Constant | -1.222 | .002 | -1.981 | -.463 |
| *Partners status by sex - cohabiting women (ref)* |  |  |  |  |
| - cohabiting men | -.217 | .035 | -.419 | -.015 |
| - not cohabiting and never married women | .900 | <.001 | .510 | 1.289 |
| - not cohabiting but previously married women | .958 | .000 | .749 | 1.167 |
| - not cohabiting and never married men | .833 | <.001 | .458 | 1.208 |
| - not cohabiting but previously married men | 1.203 | .000 | .948 | 1.459 |
|  |  |  |  |  |
| Ethnicity (non-white) | .289 | .117 | -.073 | .652 |
| *Occupation status - retired (ref)* |  |  |  |  |
| - employed | .069 | .569 | -.169 | .308 |
| - Self employed | .095 | .597 | -.258 | .448 |
| - permanently sick/disabled | 1.061 | <.001 | .657 | 1.464 |
| - Looking after home/family | .301 | .093 | -.050 | .652 |
| - other | -.109 | .738 | -.750 | .532 |
| *How much difficulty walking ¼ mile – none (ref)* |  |  |  |  |
| - some | .322 | .004 | .105 | .539 |
| - much | .433 | .003 | .146 | .720 |
| - can’t | .401 | .002 | .144 | .657 |
| Has a limiting long-standing illness | .282 | .001 | .109 | .454 |
| *Region – North or remainder of UK (ref)* |  |  |  |  |
| - South and East | .041 | .625 | -.125 | .208 |
| - Midlands | .050 | .618 | -.146 | .246 |
| *Education – less than GCSE//foreign (ref)* |  |  |  |  |
| -GSCE/A-level/equivalent | -.107 | .227 | -.281 | .067 |
| -Higher than A-level | -.186 | .042 | -.365 | -.007 |
|  |  |  |  |  |
| Age | -.010 | .056 | -.020 | .000 |
| Total wealth | -3.274E-8 | .710 | -2.060E-7 | 1.405E-7 |
| Total income | .000 | .054 | -.001 | 4.702E-6 |
| * mean N of each imputation. Imputed N varies as the number of people who have any friends varies across each imputation model. | | | | |

**Logistic regression on dichotomised UCLA scale (lonely=1), using listwise deletion. Excluding people with no friends.**

| N=5316 | **B** | **P** | **95% CI (Wald)** | |
| --- | --- | --- | --- | --- |
|  |  |  | *lower* | *upper* |
| Constant | -1.080 | .012 | -1.921 | -.238 |
| *Partners status by sex - cohabiting women (ref)* |  |  |  |  |
| - cohabiting men | -.228 | .039 | -.445 | -.012 |
| - not cohabiting and never married women | 1.024 | <.001 | .595 | 1.454 |
| - not cohabiting but previously married women | 1.010 | .000 | .791 | 1.230 |
| - not cohabiting and never married men | .898 | .000 | .494 | 1.303 |
| - not cohabiting but previously married men | 1.233 | <.001 | .956 | 1.509 |
|  |  |  |  |  |
| Ethnicity (non-white) | .249 | .270 | -.194 | .691 |
| *Occupation status - retired (ref)* |  |  |  |  |
| - employed | .089 | .486 | -.162 | .340 |
| - Self employed | .050 | .801 | -.341 | .442 |
| - permanently sick/disabled | 1.142 | <.001 | .694 | 1.589 |
| - Looking after home/family | .392 | .035 | .027 | .757 |
| - other | -.425 | .247 | -1.142 | .293 |
| *How much difficulty walking ¼ mile – none (ref)* |  |  |  |  |
| - some | .276 | .023 | .038 | .513 |
| - much | .334 | .036 | .022 | .646 |
| - can’t | .309 | .032 | .026 | .592 |
| Has a limiting long-standing illness | .325 | <.001 | .137 | .512 |
| *Region – North or remainder of UK (ref)* |  |  |  |  |
| - South and East | .021 | .818 | -.159 | .201 |
| - Midlands | .022 | .840 | -.190 | .234 |
| *Education – less than GCSE//foreign (ref)* |  |  |  |  |
| -GSCE/A-level/equivalent | -.081 | .383 | -.263 | .101 |
| -Higher than A-level | -.159 | .104 | -.350 | .033 |
|  |  |  |  |  |
| Age | -.013 | .030 | -.024 | -.001 |
| Total wealth | -3.564E-8 | .675 | -2.023E-7 | 1.311E-7 |
| Total income | .000 | .025 | -.001 | -4.224E-5 |
